# Janus Nanobullets Combine Photodynamic Therapy and Magnetic Hyperthermia to Potentiate Synergetic Anti‐Metastatic Immunotherapy

**DOI:** 10.1002/advs.201901690

**Published:** 2019-09-12

**Authors:** Zheng Wang, Fan Zhang, Dan Shao, Zhimin Chang, Lei Wang, Hanze Hu, Xiao Zheng, Xuezhao Li, Fangman Chen, Zhaoxu Tu, Mingqiang Li, Wen Sun, Li Chen, Wen‐Fei Dong

**Affiliations:** ^1^ CAS Key Laboratory of Bio Medical Diagnostics Suzhou Institute of Biomedical Engineering and Technology Chinese Academy of Sciences Suzhou 215163 China; ^2^ Department of Pharmacology Nanomedicine Engineering Laboratory of Jilin Province College of Basic Medical Sciences Jilin University Changchun 130021 China; ^3^ Department of Biomedical Engineering Columbia University New York NY 10027 USA; ^4^ State Key Laboratory of Fine Chemicals Dalian University of Technology Dalian 116024 China

**Keywords:** cancer metastasis, checkpoint blockade immunotherapy, Janus nanoparticles, magnetic hyperthermia, photodynamic therapy

## Abstract

Photodynamic therapy (PDT) is clinically promising in destructing primary tumors but ineffective against distant metastases. This study reports the use of immunogenic nanoparticles mediated combination of PDT and magnetic hyperthermia to synergistically augment the anti‐metastatic efficacy of immunotherapy. Janus nanobullets integrating chlorine e6 (Ce6) loaded, disulfide‐bridged mesoporous organosilica bodies with magnetic heads (M‐MONs@Ce6) are tailored for redox/pH‐triggered photosensitizer release accompanying their matrix degradation. Cancer cell membrane cloaking enables favorable tumor‐targeted accumulation and prolonged blood circulation time of M‐MONs@Ce6. The combination of PDT and magnetic hyperthermia has a strong synergy anticancer activity and simultaneously elicits a sequence of immunogenic cell death, resulting in synergistically tumor‐specific immune responses. When combined with anti‐CTLA‐4 antibody, the biomimetic and biodegradable nanoparticle enables the notable eradication of primary and deeply metastatic tumors with low systematic toxicity, thus potentially advancing the development of combined hyperthermia, PDT, and checkpoint blockade immunotherapy to combat cancer metastasis.

## Graphical abstract

Janus magnetic nanoparticles are designed for redox‐/pH‐triggered photosensitizer release accompanying their matrix degradation. Cancer cell membrane cloaking improves combined photodynamic therapy and magnetic hyperthermia in a remarkable anticancer manner, which is harnessed for synergistic tumor‐specific immune response to integrate with cytotoxic T lymphocyte‐associated antigen‐4 antibody for notable eradication of primary and metastatic tumors with low systematic toxicity.

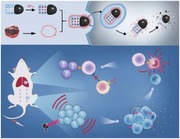

The metastatic spread of cancer cells is disastrous and often leads to ultimate death of patients.^1^ As a promising candidate for curing cancer, photodynamic therapy (PDT) has performed well and proven to be effective in realizing minimally invasive therapeutic regimen for various types of cancer.^2^ Although increasing evidence has suggested the effectiveness of PDT in cancer treatment, PDT does not entirely kill malignant cells due to the limited penetration of visible light and hypoxia at the center of tumor foci.^3^ Besides PDT, hyperthermia has been developed as an alternative or supporting remedy for cancer therapy.^4^ In addition to heat‐induced ablation of malignant cells, local hyperthermia results in a substantial improvement in tumor oxygenation and vascular perfusion by inducing blood vessel damage, which is speculated to eradicate heterogeneous tumors in hypoxic regions before the ablation of hypoxic cells.^5^ Therefore, the combination of hyperthermia and PDT may evoke a synergetic tumor response while lowering the doses of each approach, thereby minimizing side effects during cancer treatment.^6^ However, it is frustrating that such multimodal approach is not useful enough against cancer metastasis because the low penetration of excitation light and nanocarriers makes it impossible to reach deep and widespread metastatic sites.^7^

Checkpoint blockade immunotherapy that is as a clinical modality against metastasis via activating tumor‐specific T cells has exhibited unsatisfactory therapeutic efficacy in metastatic tumors due to the inefficient activation of immune responses.^8^ Substantial attention has been paid to PDT and hyperthermia‐triggered immunogenic cell death (ICD), including releasing tumor‐associated antigens, danger‐associated molecular patterns (DAMPs), and proinflammatory cytokines, which facilitate the redistribution and activation of immune effector cells with enhanced tumor‐specific T cell infiltration.^9^ To boost robust antitumor immune responses, combinational therapeutics together with multiple stimulations for amplification of immunogenicity are usually required.^10^ With these findings in mind, we hypothesize that the combination of ICD‐inducing PDT and hyperthermia was not only to facilitate therapeutic responses of primary tumors but also to provide a notable synergistic effect to eliminate distant metastatic tumors with the aid of checkpoint blockade therapy.

Magnetic mesoporous silica nanoparticles (M‐MSNs) have gained much interest for magnetic field‐mediated drug delivery, hyperthermia, and magnetic resonance (MR) imaging benefited from their intrinsic integration of magnetic and mesoporous silica compartment.^11^ While, Janus nanomaterials consist of several functional compartments in contrast with their isotropic compartments can achieve improved performance for combined therapies.^12^ In this work, an immunogenic platform based on bullet‐like Janus magnetic mesoporous organosilica nanoparticles (M‐MONs) was developed for integrated PDT, magnetic hyperthermia, and tumor‐specific immunotherapy for breast cancer. M‐MONs were embedded with chlorine e6 (Ce6), a widely used photosensitizer (PS) in PDT, at a high loading capacity. In contrast to many other nonbiodegradable silica materials, disulfide‐bridged mesoporous organosilica rods were asymmetrically grown on Fe_3_O_4_ nanospheres in our proposed system to achieve tumor microenvironment (dual redox/pH) responsive drug release and reduce long‐term in vivo toxicity due to matrix‐mediated degradation. The obtained M‐MON@Ce6 nanoparticles (NPs) were further cloaked with breast cancer cell membrane to yield CM@M‐MON@Ce6, which achieved homologous tumor‐targeted accumulation and prolonged blood circulation time. **Scheme** 1 illustrates our strategy of employing CM@M‐MON@Ce6 not only to develop two therapeutic modalities (PDT and magnetic hyperthermia) to achieve the synergistic regression of primary tumors but also to effectively trigger an antitumor immune response, which was combined with cytotoxic T lymphocyte‐associated antigen‐4 (CTLA‐4) checkpoint blockade to suppress distant metastasis. Our unique system was motivated by a two‐way mechanistic interaction to combine (i) a Janus nanobullet that facilitates the combination of PDT and magnetic hyperthermia with a marked eradication efficacy in primary tumor and (ii) checkpoint blockade immunotherapy that synergistically integrates ICDs after combined hyperthermia and PDT for distant metastasis inhibition.

**Scheme 1:**
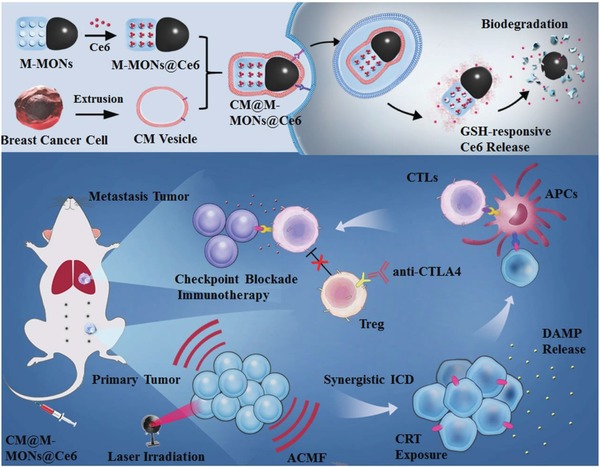
A schematic of the synthetic procedure for the cancer cell membrane‐cloaked Ce6‐loaded Janus magnetic mesoporous organosilica nanoparticles (CM@M‐MON@Ce6) and their application for combined PDT and magnetic hyperthermia to further potentiate a CTLA‐4 blockade to enhance synergistic antitumor immunity in combating cancer metastasis.

A modified sol–gel method was utilized to prepare Janus‐structure‐like nanobullets with spherical Fe_3_O_4_ NPs as the head and a disulfide‐bridged mesoporous silica framework as the body. Electron microscopy images of the M‐MONs clearly revealed a uniform anisotropic morphology and overall dimensions of 250 nm × 100 nm (**Figure** 1a and Figure S1a, Supporting Information). Since the disulfide bonds in the framework of the M‐MONs tend to be cleaved in reductive conditions,^13^ the silica portion gradually degraded and eventually collapsed into fragments in simulated GSH solutions within 5 d (Figure 1b and Figure S1b,c, Supporting Information). The M‐MONs also exhibited a good magnetic response with magnetization saturation at 63 emu g^−1^ (Figure 1c), showing superparamagnetic properties similar to those of Fe_3_O_4_ nanospheres. As a result, the M‐MOMs demonstrated excellent and stable magnetic‐thermal performance after 20 min of exposure to an alternating current magnetic field (ACMF) (Figure 1d). The M‐MONs exhibited a large surface area, pore volume, and average pore diameter of 721.5 m^2^ g^−1^, 0.57 cm^3^ g^−1^, and 3.8 nm, respectively (Figure 1e), which allowed for the sufficient loading of a large variety of hydrophobic PSs.

**Figure 1:**
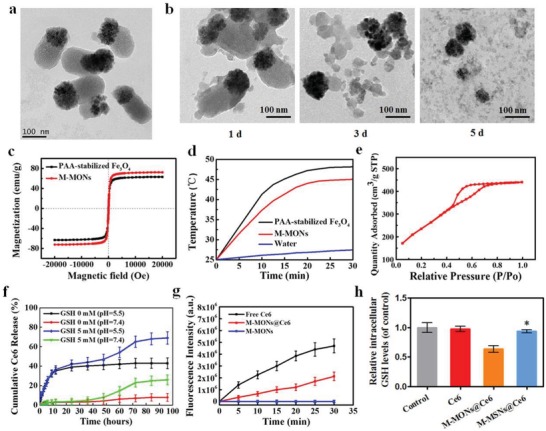
Characterization of M‐MON@Ce6. a) TEM images of M‐MONs. b) M‐MONs were immersed in 5 × 10^−3^ m GSH solution for 1, 3, and 5 d. c) The magnetization curve, d) temperature–time curves, and e) N_2_ sorption isotherms of M‐MONs. f) Drug release profiles of Ce6@M‐MONs in 0 and 5 × 10^−3^ m GSH (pH = 7.4 and 5.5). g) Time‐dependent SOSG fluorescence in Ce6 and M‐MON@Ce6 solutions. h) Intracellular GSH levels of MCF‐7 cells after treatment with M‐MON@Ce6 for 12 h. The data are presented as the mean ± S.D. (*n* = 3). *p < 0.05 versus the M‐MON@Ce6 group.

To use M‐MONs for PDT applications, Ce6 was selected as a model PS and loaded into amino‐functionalized M‐MONs at a concentration of 10.8 wt% (Figure S1d, e, Supporting Information). The release of Ce6 in normal phosphate buffer saline (PBS) solution was slow. In contrast, Ce6 release was substantially accelerated under reductive conditions at 24–72 h due to the matrix degradation of the M‐MONs (Figure 1f). In addition, the drug release content of M‐MON@Ce6 also increased to some extent in the low pH conditions due to faster and more complete dissociation of Ce6. Importantly, compared with the drug release profiles of M‐MON@Ce6 in a GSH‐free solution at pH 5.5, the release rates of Ce6 were much faster in the 5 × 10^−3^ m glutathione (GSH) solution at the same pH. To further demonstrate the advantages of M‐MONs for degradation and drug delivery, Janus M‐MSNs were synthesized as a nonbiodegradable control,[qv: 12a] and their characteristics were similar to those of M‐MONs (Figure S2, Supporting Information). As expected, M‐MSNs exhibited less drug release than M‐MONs due to their nondegradable response in GSH solution (Figure S3, Supporting Information). Given that the tumor microenvironment is acidic and reductive,^14^ the dual redox and pH‐responsive behavior of M‐MONs is desirable for drug release with considerably reduced toxicity to vital organs and decreased demand for medical intervention. Furthermore, M‐MON@Ce6 under light exposure showed effective singlet oxygen (SO) production (Figure 1g), which was slightly less than that of free Ce6 at the same concentration. The less SO production of M‐MON@Ce6 might be attributed to the quenching effect and insufficient Ce6 release in the initial process, which could be recovered after 24 h of GSH incubation (Figure S1f, Supporting Information). Importantly, M‐MONs@Ce6 induced a greater reduction in intracellular GSH levels than M‐MSN@Ce6 (Figure 1h), which might be attributed to disulfide bridges in the framework of M‐MONs that could consume GSH.^15^ Since a high concentration of GSH in cancer cells substantially reduces the efficiency of PDT, Ce6‐loaded M‐MONs were expected to be more efficient in PDT than free Ce6 due to their GSH depletion ability.

To enhance the physiological stability of M‐MON@Ce6 and provide homologous targeting and immune‐evading properties,^16^ M‐MON@Ce6 molecules were cloaked with cancer cell‐biomimetic vesicles (CMs) derived from MCF‐7 breast cancer cells according to our previously reported method.[qv: 12a] Biomimetic CM@M‐MON@Ce6 molecules with a uniform layer containing membrane protein components were verified by transmission electron microscopy (TEM) images (**Figure** 2a), zeta potential (Figure 2b), and particle size changes (Figure S4a, Supporting Information). Additionally, sodium dodecyl sulfate polyacrylamide gel electrophoresis (SDS‐PAGE) of CM@M‐MON@Ce6, cancer cell membranes, and CMs was conducted (Figure 2c). M‐MON@Ce6 with a cell membrane surface coating was dispersed in PBS without any aggregation over 7 d of incubation, suggesting their excellent stability in aqueous solution (Figure S4b, Supporting Information).

**Figure 2:**
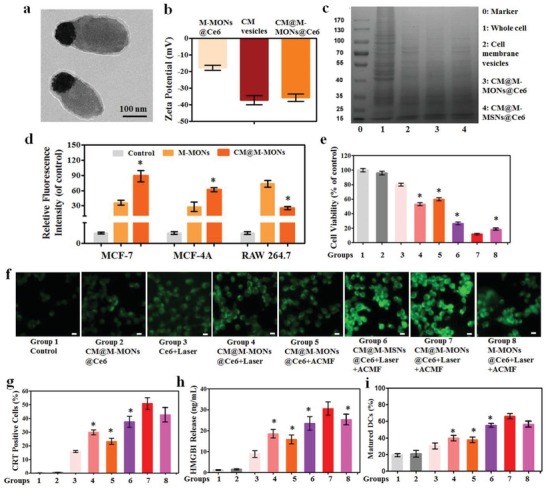
Combined PDT and magnetic hyperthermia by CM@M‐MON@Ce6 in vitro. a) TEM images, b) zeta potential, and c) SDS‐PAGE protein analysis of CM@M‐MON@Ce6. d) The relative fluorescence intensity of MCF‐7, MCF‐10A, and RAW264.7 cells after incubation with CM@FITC‐M‐MONs for 6 h. The data are presented as the mean ± S.D. (*n* = 3). *p < 0.05 compared with the M‐MON group. e–h) MCF‐7 cells incubated with CM@M‐MON@Ce6 (12.5 µg mL^−1^) for 2 h, followed by a 20 min exposure to an ACMF or/and 5 min of exposure to laser irradiation with a 20 min exposure to an ACMF. e) Cell viability after 24 h of exposure. f) Intracellular reactive oxygen species (ROS) fluorescence images after 6 h of exposure; the scale bars indicate 10 µm. g) The percentage of CRT‐positive cells and h) the amount of released HMGB1 after 24 h of exposure. i) The percent of mature DCs (CD11c^+^CD80^+^CD86^+^) in BMDCs after co‐incubation with different treated MCF‐7 cells for 24 h. The data are presented as the mean ± S.D. (*n* = 3). *p < 0.05 compared with the CM@M‐MON@Ce6+Laser+ACMF group. Group description of (e), (g), (h), and (i) was the same as illustrated in (f).

After demonstrating the good biocompatibility and endosome‐mediated endocytosis of M‐MONs in MCF‐7 cells (Figure S5, Supporting Information), we observed a high degree of intracellular colocalization between M‐MONs and CMs in endosomes after 1 h incubation (Figure S6, Supporting Information), while M‐MONs partially escaped from endosomes to the cytoplasm after 6 h of cellular uptake. Next, we compared the effect of CM cloaking on the targeting ability of M‐MONs in MCF‐7 and MCF‐10A cells and in RAW264.7 murine macrophages through flow cytometry analysis (Figure 2d) and fluorescence images (Figures S7–S9, Supporting Information). Compared to M‐MONs, CM@M‐MONs exhibited an increased intensity in both MCF‐7 and MCF‐10A cells, while they showed decreased signals in macrophages. The immune escape effects might be attributed to the fact that high expression of cell membrane protein CD47 present on the CM@MONs@Ce6 (Figure S10, Supporting Information) related to homotypic cell adhesion, therefore inhibiting the phagocytic uptake of macrophages.[qv: 16a] Importantly, a significantly higher fluorescence intensity was observed in MCF‐7 cells than in MCF‐10A cells in the CM@M‐MON group, but not in the M‐MON group. These well‐known homologous targeting effects could be caused by adhesion molecules on the breast cell membrane due to a higher affinity for their source cells.^17^ Additionally, intracellular Ce6 fluorescence, which indicated the GSH/pH‐sensitive release of Ce6 within acidic lysosomes, was significantly enhanced with prolonged incubation time in CM@M‐MON@Ce6‐treated cells rather than in CM@M‐MSN@Ce6‐treated cells (Figure S11, Supporting Information). Collectively, cancer cell membrane‐cloaked M‐MON@Ce6 exhibited homologous targeting and immune escape effects with tumor microenvironment‐responsive PS release.

We sought to investigate the combined PDT and magnetic hyperthermia mediated by CM@M‐MON@Ce6. First, the PDT efficiency of CM@M‐MON@Ce6 was evaluated (Figure S12a, Supporting Information). The light‐triggered cancer cell killing efficiency of CM@M‐MON@Ce6 was significantly greater than that of free Ce6 and CM@M‐MSN@Ce6, likely owing to the decreased GSH level and increased Ce6 release in MCF‐7 cells. Then, the efficiency of magnetic hyperthermia was investigated through preincubation MCF‐7 cells with CM@M‐MON@Ce6 for 6 h, followed by exposing the cells to an ACMF for 20 min (Figure S12b, Supporting Information). CM@M‐MON@Ce6 exhibited a dose‐dependent effect on magnetic hyperthermia, whereas no difference in cell viability was observed after incubating with CM@M‐MON@Ce6 or CM@M‐MSN@Ce6, respectively. Considering the relatively effective PDT and magnetic hyperthermia, the synergistic effect of combinatorial PDT and magnetic hyperthermia facilitated by CM@M‐MON@Ce6 was further examined (Figure 2e). Compared with PDT alone (CM@M‐MON@Ce6 plus laser) and magnetic hyperthermia alone (CM@M‐MON@Ce6 plus ACMF), the combined therapy (CM@M‐MON@Ce6 plus laser and ACMF) was the most effective in inducing cancer cell death in a synergistic manner. The combined therapeutic efficiency of CM@M‐MON@Ce6 was markedly enhanced, further demonstrating the advantages of CM cloaking and biodegradable silica compartments in the unique system. Owing to the high reactivity, SO, with an extremely short half‐life within tens of nanoseconds in cytoplasm, was transformed to ROS and difficult to be directly detected.^18^ Thus, the intracellular ROS was detected as an alternative between different groups (Figure 2f and Figure S13, Supporting Information). A significant enhancement in ROS signals was detected in the CM@M‐MON@Ce6 plus laser and ACMF group compared to other control groups, which demonstrated that the combination of PDT and magnetic hyperthermia increased intracellular ROS levels in MCF‐7 cells and thus enhanced the cytotoxicity. Similar to the trends in cell viability and ROS generation, we revealed that CM@M‐MON@Ce6 induced the highest apoptotic rate in MCF‐7 cells (Figure S14, Supporting Information), indicating that the combination of PDT and magnetic hyperthermia is an effective ROS‐mediated cell death inducer in vitro.

PDT and magnetic hyperthermia have been reported to cause immunogenic cell death, resulting in calreticulin (CRT) exposure and chromatin‐binding protein high mobility group B1 (HMGB1) release as classic indicators of ICD.^19^ After exposure or release, these danger molecules promote the recognition and processing of antigen‐presenting cells, followed by T lymphocyte‐mediated anticancer immunity.^20^ Therefore, we investigated the ability of CM@M‐MON@Ce6 with laser and ACMF stimuli to induce immunogenic phenotypes in MCF‐7 cells. As shown in Figure 2g, significantly increased amounts of CRT‐positive cells were observed after treating cells with three types of Ce6‐loaded NPs combined with laser and ACMF stimuli, while the NPs without irradiation or ACMF did not yield different results than the control group. Importantly, CM@M‐MON@Ce6 with the combined laser and ACMF stimuli induced greater amounts of CRT‐positive cells than CM@M‐MON@Ce6 with the laser or ACMF stimuli alone. In addition to CRT exposure, CM@M‐MON@Ce6‐mediated PDT and magnetic hyperthermia also induced the greatest amount of HMGB1 release during anticancer therapy (Figure 2h). To further evaluate immunological effects of CM@M‐MON@Ce6‐mediated PDT and magnetic hyperthermia, we investigated ICD‐induced dendritic cell (DC) maturation in vitro. It was also found that CM@M‐MON@Ce6 with the combined laser and ACMF stimuli could greatly promote in vitro DC maturation than CM@M‐MON@Ce6 with laser or ACMF stimuli alone (Figure 2i). Overall, these results suggest that combined PDT and magnetic hyperthermia of CM@M‐MON@Ce6‐induced synergistic ICD and DC maturation in MCF‐7 cells could be an effective anticancer vaccine in the in vivo model.

Having demonstrated the combined PDT and magnetic hyperthermia function of CM@M‐MON@Ce6 in our in vitro experiments, we first investigated the tumor‐specific imaging and biodistribution of CM@M‐MON@Ce6 in MCF‐7 tumor‐bearing nude mice. A significant concentration‐dependent darkening effect by CM@M‐MON@Ce6 samples was also found in T2‐weighted MR images due to the dipolar interaction of the protons in water with the magnetic component of CM@M‐MON@Ce6 (Figure S15a, Supporting Information). The corresponding transverse relaxivity (r2) value of CM@M‐MON@Ce6 was 115.6 mm ^−1^ s^−1^ (Figure S15b, Supporting Information). The maximum T2‐weighted MR imaging signal enhancement of the tumor site was achieved at 6 h postinjection and decreased at 24 h in only the CM@M‐MON@Ce6‐treated mice due to an efficient CM‐mediated tumor homing effect (Figure S15c, Supporting Information). Given the immune escape property of cell membrane, the blood circulation half‐life of CM@M‐MON@Ce6 (5.6 h) was 4.7 times greater than that of M‐MON@Ce6 (1.2 h) (**Figure** 3a). The biodistribution results also revealed that cell membrane camouflaging enhanced tumor accumulation and decreased the enrichment in the reticuloendothelial system, including the liver and spleen (Figure 3b). Collectively, our findings provided direct evidence that CM‐cloaked M‐MON@Ce6 with effective T2‐weighted MR contrast performance, prolonged blood circulation time, and immune‐evasive ability is promising for homologous tumor‐specific targeting and drug delivery.

**Figure 3:**
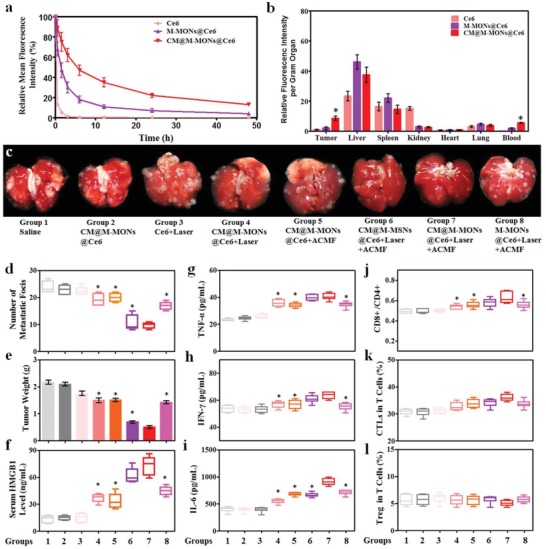
Anti‐tumor effects and immune responses after combined PDT and magnetic hyperthermia with CM@M‐MON@Ce6. a) The blood circulation time, and b) biodistribution of CM@M‐MON@Ce6 in MCF‐7 tumor‐bearing mice. c) Representative images of lung tissues with observable metastatic nodules. d) The number of pulmonary metastatic nodules and e) primary tumor weights of 4T1 tumor‐bearing mice from each group over 21 d. After 5 d of combined PDT and magnetic hyperthermia, serum and primary tumor tissue were collected for the analysis of f) HMGB1, g) TNF‐α, h) IFN‐γ, and i) IL‐6 levels in serum and for the analysis of j) the ratios of CD8+T cells/CD4+T cells, k) CTL content, and l) Treg content in the primary tumor tissues. The data are presented as the mean ± S.D. (*n* = 5, *p < 0.05 compared with the CM@M‐MON@Ce6+Laser+ACMF group).

Next, we investigated the efficacy of CM@M‐MON@Ce6 for combined PDT and magnetic hyperthermia in an orthotropic MCF‐7 nude mouse tumor model. As shown in Figure S16 (Supporting Information), CM@M‐MON@Ce6 did not show a substantial effect on tumor growth, and free Ce6 with the laser treatment also did not exhibit a noticeable effect on tumor growth due to inefficient PDT. Tumor growth in mice treated with CM@M‐MON@Ce6 and the laser or with ACMF was partially delayed. Importantly, the tumors in the combined treatment group (CM@M‐MON@Ce6+Laser+ACMF) showed the slowest growth rate and the greatest tumor inhibition rate at the end of the treatment. As expected, the tumor inhibition rates in the CM@M‐MON@Ce6+Laser+ACMF group were notably greater than those in the corresponding CM@M‐MSN@Ce6+Laser+ACMF and M‐MON@Ce6+Laser+ACMF groups, consistent with tumor microenvironment‐responsive Ce6 release and CM‐mediated tumor‐specific targeting properties, respectively. It is worth to note that we applied ACMF treatment before PDT treatment because of ACMF‐induced hyperthermia induced blood vessel damage and a substantial improvement in tumor oxygenation,^5^ which is speculated to facilitate eradicating heterogeneous tumors in hypoxic regions during PDT. Furthermore, the body weight, serum biochemistry, and organ histology of the liver, spleen, kidneys, heart, and lungs in all treated mice showed negligible changes (Figures S17–S19, Supporting Information), suggesting the good biosafety profile of M‐MON@Ce6‐based treatments.

Encouraged by the synergistic effects of CM@M‐MON@Ce6‐mediated combined PDT and magnetic hyperthermia, we further evaluated the therapeutic efficacy and tumor‐specific immune responses of 4T1 cell‐derived CM‐cloaked M‐MON@Ce6 using an orthotropic 4T1 BALB/c mouse tumor model. 4T1 tumor‐bearing mouse model was used in this case, because 4T1 is an aggressive cell line, which could induce 100% pulmonary metastasis after 3 weeks of orthotopical implantation. Similar to the results in the MCF‐7 model, compared with mice treated with CM@M‐MON@Ce6+Laser, CM@M‐MON@Ce6+ACMF, CM@M‐MSN@Ce6+Laser+ACMF, and M‐MON@Ce6+Laser+ACMF, mice treated with CM@M‐MSN@Ce6+Laser+ACMF exhibited significantly delayed growth and decreased weights of the primary tumors (Figure 3e and Figure S20, Supporting Information). Notably, CM@M‐MON@Ce6+Laser+ACMF only partially decreased the number of pulmonary metastatic nodules (Figure 3c,d and Figure S20d, Supporting Information), consistent with the evidence that the combination of PDT and magnetic hyperthermia locally inhibited tumor growth instead of remarkably affecting tumor metastasis. Such performance might be attributed to the fact that those 4T1 cell‐derived CM@M‐MONs@Ce6 may exhibit similar biodistribution manner in this model due to the homologous targeting effects demonstrated by us and other groups.^16^

Extensive evidence highlighted that PDT and hyperthermia induced immunological responses through the exposure and release of danger molecules.^21^ However, it has been shown that combined PDT and magnetic hyperthermia might synergistically induce tumor‐specific immunity. As shown in Figure 3f, the CM@M‐MSN@Ce6+Laser+ACMF treatment significantly increased HMGB1 release in orthotopic 4T1 tumor tissues, which is a result that is consistent with our in vitro findings (Figure 2h). The production of cytokines, including tumor necrosis factor‐α (TNF‐α), interferon‐γ (IFN‐γ), and interleukin‐6 (IL‐6), which are important biomarkers that indicate antitumor immunity induced by PDT and magnetic hyperthermia, in the combination group was markedly greater than that in the control and monotherapy groups (Figure 3g–i). After the initial indication of a systemic immune response, we further profiled infiltrating leukocytes in the primary tumors. Compared with CM@M‐MON@Ce6+Laser and CM@M‐MON@Ce6+ACMF treatments, the CM@M‐MSN@Ce6+Laser+ACMF treatment increased the ratio of CD8^+^ T cells to CD4^+^ T cells in the mice (Figure 3j). Cytotoxic T lymphocyte (CTL) recruitment to the primary tumors in the combined treatment group compared with that in the monotherapy groups increased (Figure 3k). Correspondingly, the percentage of regulatory T cells (Treg) exhibited a slight decrease in the CM@M‐MSN@Ce6+Laser+ACMF group compared with the other groups (Figure 3l). These findings demonstrated that combined PDT and magnetic hyperthermia synergistically induced tumor‐specific immune responses, including the activation of immune effector cells and enhanced tumor‐specific T cell infiltration. Therefore, such synergistic immune responses induced with the assistance of an anti‐CTLA‐4 checkpoint inhibitor are considered an effective approach to inhibit tumor metastasis.

To determine whether the antitumor immune response triggered by the combination of PDT and magnetic hyperthermia sensitizes tumors to checkpoint blockade therapy, we investigated the antitumor activity and antimetastatic effect of CM@M‐MON@Ce6+Laser+ACMF combined with anti‐CTLA‐4 (α‐CTLA‐4) on an orthotropic 4T1 BALB/c mouse tumor model (**Figure** 4a). Although anti‐CTLA‐4 alone did not delay 4T1 tumor progression and metastasis, the combination of anti‐CTLA‐4 with PDT and/or magnetic hyperthermia showed significantly different primary tumor growth inhibition efficiencies and pulmonary metastatic suppression activities than the combined treatment without immunotherapy (Figure 4b–f and Figure S21a, Supporting Information). Notably, CM@M‐MON@Ce6+Laser+ACMF combined with the anti‐CTLA‐4 treatment completely eradicated the primary 4T1 tumors and prevented the development of the pulmonary metastatic nodules, indicating that the combined treatment was markedly better than either CM@M‐MON@Ce6+Laser+α‐CTLA‐4 or CM@M‐MON@Ce6+ACMF++Laser+α‐CTLA‐4 andion. Notably, CM@M‐MON@Ce6+Laser+ACMF combined with the anti‐CTLA‐4 treatment c (Figure 4g), CTL activation (Figure 4h), Treg inhibition (Figure 4i), and proinflammatory cytokine production (Figure S21b–d, Supporting Information)were observed in the CM@M‐MON@Ce6+ACMF+α‐CTLA‐4 group compared with the monotherapies combined with α‐CTLA‐4 groups. These immune responses amplified by the CTLA‐4 inhibitor led to a notable systemic therapeutic outcome to effectively suppress the growth of primary and metastatic tumors. In light of this, the enhanced anti‐tumor metastasis induced by the combination of therapy with anti‐CTLA‐4 checkpoint inhibitor might be explained by the synergistic anti‐cancer immune response triggered by PDT and hyperthermia‐induced ICDs including releasing HMGB1 and proinflammatory TNF‐α, IFN‐γ, and IL‐6, which facilitate the DCs maturation, enhanced CTLs infiltration, and Treg inhibition. Encouragingly, the body weight, blood biochemistry, and organ histopathology in all treated mice were not different than those in the untreated healthy mice (Figures S22–S24, Supporting Information), indicating that systemic toxicity was not noticeably induced by our combined approach. Although there are already many reported delivery platforms based on magnetic MSNs for synergistic tumor therapy,^22^ almost all the platforms as core–shell type, which may reduce the outcomes of synergistic tumor therapy due to the interferes of each compartment. Our M‐MONs with GSH‐responsive degradability exhibited faster Ce6 release, more DAMP release, and stronger anti‐metastatic effect with the combination of checkpoint blockade therapy.

**Figure 4:**
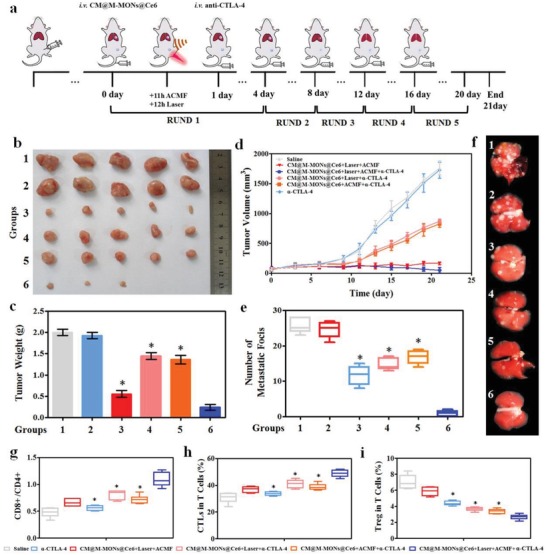
The synergistic effects of CM@M‐MON@Ce6‐mediated PDT and magnetic hyperthermia in combination with anti‐CTLA4 checkpoint blockade. a) A schematic of CM@M‐MON@Ce6‐mediated PDT and magnetic hyperthermia and anti‐CTLA4 checkpoint blockade combined therapy. 4T1 tumor‐bearing mice were randomly divided into the following eight groups: saline (group 1), α‐CTLA‐4 (group 2), CM@M‐MON@Ce6+Laser+ACMF (group 3), CM@M‐MON@Ce6+Laser+α‐CTLA‐4 (group 4), CM@M‐MON@Ce6+ACMF+α‐CTLA‐4 (group 5), and CM@M‐MON@Ce6+Laser+ACMF+α‐CTLA‐4 (group 6). b) Representative tumor images, c) tumor weights, d) tumor volumes, e) number of pulmonary metastatic nodules, and f) representative images of lung tissues with observable metastatic nodules of 4T1 tumor‐bearing mice from each group over 21 d. After 9 d of combined PDT, magnetic hyperthermia, and immune checkpoint therapy, serum and primary tumor tissue were collected for the analysis of g) the ratios of CD8+T cells/CD4+T cells, h) CTL content, and i) Treg content in the metastatic tumor tissues. The data are presented as the mean ± S.D. (*n* = 5, *p < 0.05 compared with the CM@M‐MON@Ce6+Laser+ACMF group).

In conclusion, we developed bullet‐like Janus magnetic mesoporous organosilica nanoparticles with a cancer cell membrane coating and PS loading as a multifunctional platform for combined PDT‐magnetic hyperthermia, which synergistically boosted an antitumor immune response to promote metastatic suppression. Compared with the structure of traditional Janus M‐MONs, the well‐defined mesoporous structure containing a disulfide‐bridged organosilica framework exhibited improvements in tumor microenvironment‐responsive controlled Ce6 release, GSH depletion, and ROS augmentation. Notably, the cancer cell membrane cloaking exhibited homologous targeting and immune escape behavior in breast cancer cells, thus facilitating targeted MR imaging and increased blood circulation time. PDT and magnetic hyperthermia with CM@M‐MON@Ce6 effectively inhibited tumor growth in orthotropic MCF‐7 and 4T1 tumor‐bearing mouse models. More importantly, both PDT and magnetic hyperthermia contributed to ICD, including CRT exposure and HMGB1 release, which markedly enhanced the synergistic generation of an antitumor immune response. Therefore, CM@M‐MON@Ce6‐mediated PDT and magnetic hyperthermia combined with anti‐CTLA‐4 suppressed the growth of not only primary tumors but also metastatic tumors with no noticeable systemic toxicity. Our biomimetic and biodegradable nanobullets showed promising potential for the combination of PDT, magnetic hyperthermia, and checkpoint blockade therapy, providing a promising and safe strategy for the cancer treatment, particularly for the treatment of advanced cancer with existing metastasis.

## Conflict of Interest

The authors declare no conflict of interest.

## Supporting information

Supplementary
