# Supplementary material for: Janus Nanobullets Combine Photodynamic Therapy and Magnetic Hyperthermia to Potentiate Synergetic Anti‐Metastatic Immunotherapy
Source: Adv Sci (Weinh). 2019 Sep 12;6(22):1901690. doi: 10.1002/advs.201901690 (PMC6864517; doi:10.1002/advs.201901690)
Supplement: Supplementary file 1 — Supplementary [file ADVS-6-1901690-s001.pdf]

Copyright WILEY-VCH Verlag GmbH & Co. KGaA, 69469 Weinheim, Germany, 2019.

ADVANCED SCIENCE
Open Access

Supporting Information

for *Adv. Sci.,* DOI: 10.1002/advs.201901690

Janus Nanobullets Combine Photodynamic Therapy and Magnetic Hyperthermia to Potentiate Synergetic Anti-Metastatic Immunotherapy

Zheng Wang, Fan Zhang, Dan Shao, Zhimin Chang, Lei Wang, Hanze Hu, Xiao Zheng, Xuezhao Li, Fangman Chen, Zhaoxu Tu, Mingqiang Li, Wen Sun,* Li Chen, and Wen-Fei Dong**

Copyright WILEY-VCH Verlag GmbH & Co. KGaA, 69469 Weinheim, Germany, 2019.

Supporting Information

Zheng Wang, Fan Zhang, Dan Shao, Zhimin Chang, Lei Wang, Hanze Hu, Xiao Zheng, Xuezhao Li, Fangman Chen, Zhaoxu Tu, Mingqiang Li, Wen Sun,* Li Chen, and Wen-fei Dong**

Dr. Z. Wang, F. Zhang, Dr. D. Shao, Z. Chang, F. Chen, Prof. W. F. Dong
CAS Key Laboratory of Bio Medical Diagnostics, Suzhou Institute of Biomedical Engineering and Technology, Chinese Academy of Sciences, Suzhou 215163, China.
Dr. D. Shao, H. Hu, Z. Tu, Prof. M. Li
Department of Biomedical Engineering, Columbia University, New York, NY 10027, USA
L. Wang, X. Li, Prof. W. Sun
State Key Laboratory of Fine Chemicals, Dalian University of Technology, Dalian 116024, China.
Dr. D. Shao, F. Zhang, Dr. X. Zheng, Prof. L. Chen
Department of Pharmacology, Nanomedicine Engineering Laboratory of Jilin Province, College of Basic Medical Sciences, Jilin University, Changchun 130021, China.

Correspondence authors: wenfeidong@sibet.ac.cn; stanauagate@outlook.com; sunwen@dlut.edu.cn

Experimental Section

Materials: Tetraethyl orthosilicate (TEOS), cetyltrimethyl ammonium bromide (CTAB), bis[3-(triethoxysilyl)propyl]tetrasulfide (BTESPT), iron (III) chloride anhydrous, polyacrylic acid (PAA, molecular weight≈1800), diethylene glycol, 3-aminopropyl triethoxysilane (APS), fluorescein isothiocyanate (FITC), and sulforhodamine B (SRB) were purchased from Sigma-Aldrich. Chlorin e6 (Ce6) was purchased from J&K Scientific, Ltd. Sodium hydroxide (NaOH), ammonium

hydroxide (NH_4OH, 28%), triethylamine (TEA), ammonium nitrate (NH_4NO_3), anhydrous ethanol, and hydrochloric acid were purchased from Beijing Chemical Reagent Co. LysoTracker Red DND-99, DiD, 4',6-diamidino-2-phenylindole (DAPI), and 2',7'-dichlorodihydrofluorescein diacetate (DCFH-DA) were obtained from Invitrogen. Singlet oxygen sensor green (SOSG) was obtained from Thermo Fisher Scientific. A GSH kit was obtained from Cell Signaling Technology. Dulbecco's modified Eagle medium-high glucose (DMEM) and fetal bovine serum (FBS) were obtained from Gibco. Penicillin, streptomycin and trypsin were purchased from Beyotime Institute of Biotechnology Co. All reagents were commercially available products of analytical grade purity and were used without further purification.

*Synthesis and modification of M-MON*s: Fe_3O_4 magnetic spheres were synthesized according to our previously reported method.[1] Then, an aqueous solution of Fe_3O_4 (10 mg) was added to 20 mL of suspended CTAB (100 mg) solution under ultrasonication. Then, 0.5 mL of NH_4OH was injected into the solution at 40 °C, followed by the addition of 20 μL of TEOS and 5 μL of BTESPT. After 30 min of mechanical stirring, the solution was collected by a magnetic field and washed with ethanol three times. To extract CTAB, as-synthesized M-MONs were dispersed in 100 mL of ethanol solution containing 10 mg/mL NH_4NO_3 and refluxed for 6 h. The obtained M-MONs were collected, purified and used for the further experiments. The preparation of M-MSNs was conducted in accordance with our previous reports.[2] Then, FITC-labeled MSNs fabricated according to our previously reported method.[3] Briefly, FITC-APS conjugation was conducted in advance by stirring FITC in an

ethanol-APS solution (10% w/w) and refluxed with MSNs in the dark overnight. Next, the obtained NPs were dispersed in dimethylformamide (DMF) solution containing succinic anhydride with the aid of TEA, and negatively charged FITC-labeled NPs were purified and stored in the dark for the further experiments.

Characterization: TEM images of the NPs were obtained using a Hitachi model H-7650 transmission electron microscope (Hitachi, Ltd., Tokyo, Japan) at an accelerating voltage of 100 kV. The morphologies of the NPs were further analyzed using a field-emission scanning electron microscope (FE-SEM, S4800, Hitachi). The specific surface area was determined by the Brunauer-Emmett-Teller (BET) method, and the pore size distributions were determined by the Barrett-Joyner-Halenda (BJH) method. Magnetic measurements were carried out using a TDM-B vibrating sample magnetometer (VSM) at 300 K. UV-Vis absorption spectra were obtained on a U-3310 spectrophotometer (Hitachi, Japan). The size and zeta potential of the NPs were determined by a Malvern Zeta sizer Nano ZS instrument. SDS-PAGE was used to characterize the protein composition of the NPs. Stability experiments were performed by analyzing the size of the NPs in FBS for 7 days. The production of singlet oxygen by the NPs was analyzed by a SOSG assay according to the manufacturer's protocol, the M-MON@Ce6 solutions were exposed to light with a wavelength of 660 nm for 5 min (0.15 W/cm^2). The magnetic hyperthermia property of the M-MONs@Ce6 (1 mg mL^{-1}) with the frequency of 325 Oe, 262 kHz were investigated using the fibers optic probe in the solution.

Degradation and drug release: The degradation of different MSNs was evaluated by incubating MSNs (100 μg/mL) with 5 mM GSH solution at 37 °C under constant rotation. At predetermined timepoints, the solution was analyzed by TEM and DLS, and the Si concentration of surfactant after centrifugation was measured by inductively coupled plasma mass spectrometry (ICP-MS) (Xseries II, Thermo Scientific, USA). For Ce6 loading, MSNs were functionalized with amino groups through a postgrafting method.[30] Then, the MSNs (1 mg/mL) were mixed with 1 mg/mL Ce6 solution in dimethylsulfoxide (DMSO) for 12 h, yielding MSN@Ce6 by centrifugation to remove unbound Ce6. Drug loading content was determined by analyzing the UV-vis absorbance at a wavelength of 403 nm according to the following Equation:

Ce6-loading content (%) = Mass of Ce6 in MONs@Ce6/Mass of MONs@Ce6. To investigate the Ce6 release profile, a solution of MSN@Ce6 was dialyzed against water or 5 mM GSH solution with different pH values (7.4 and 5.5) at 37 °C. The amounts of Ce6 released at different timepoints were measured and calculated.

Preparation and characterization of CM@M-MON@Ce6: The 4T1 murine BC cell line, MCF-7 human BC cell line, MCF-10A human mammary epithelial cell line and RAW264.7 murine macrophage cell line were originally obtained from American Type Culture Collection (ATCC) and maintained in DMEM supplemented with 10% FBS and antibiotic solution. To obtain cancer cell membrane-derived vesicles, MCF-7 and 4T1 cells were collected, hypotonically lysed and centrifuged, and then an extrusion approach and sonication were employed according to our previously

reported study.[4] DiD-labeled vesicles were prepared by prestaining and washing the collected cancer cells before hypotonic lysis. The obtained cancer cell membrane-derived vesicles were mixed with M-MON@Ce6 solutions. The resulting mixtures were sonicated with heating for 5 min and subsequently extruded through 400 nm polycarbonate membranes to obtain cancer cell membrane-cloaked Ce6-loaded M-MONs (CM@M-MON@Ce6). The final products were stored at 4 °C until further use.

The marker protein CD47 of the BC cells, BC cell membranes and CM@M-MON@Ce6 from both MCF-7 cells and 4T1 cells was identified by western blot analysis. Proteins from different samples quantified by BCA protein assay kit to ensure that identical amounts of protein from each sample was loaded. The samples were separated on 10% SDS-PAGE and transferred onto polyvinylidene fluoride (PVDF) membranes. After blocked by TBS buffer (150 mM NaCl, 10 mM Tris, pH 7.4) containing 5% nonfat milk, the blots were incubated with a primary antibody at 4 °C overnight and secondary antibody for 1 h at room temperature. The primary antibody of anti-CD47 (1:1000, ab175388) and the secondary antibody goat anti-rabbit IgG (1:2000, ab205718) were obtained from Abcam. The blots were visualized by super ECL.

In vitro cell experiments: For the cell toxicity assay, cells were seeded into 96-well plates at a density of 5×10^3 cells/well overnight. After the cells were incubated with a series of NP concentrations with or without irradiation for different periods of time, a standard sulforhodamine B (SRB) assay was used to analyze cell

viability relative to untreated cells. For the apoptosis assay, cells were seeded into 6-well plates at a density of 5 × 10^4 cells/well overnight. After the cells were incubated with a series of NP concentrations with or without irradiation for different periods of time, the cells were collected and stained using an FITC-Annexin V/dead cell apoptosis kit according to the manufacturer's protocol and quantitatively analyzed by flow cytometry (FACS, Becton-Dickinson Biosciences, Drive Franklin Lakes, USA). All statistical analyses were based on three independent experiments.

To analyze endocytosis of the NPs, cells were seeded in a 24-well plate at a density of 3 × 10^4 cells/well overnight. Then, the cells were incubated with FITC-labeled NPs or DiD-stained cell membrane-cloaked FITC-labeled NPs at a final NP concentration of 12.5 μg/mL. After different incubation times, the cells were stained with LysoTracker Red DND-99 and DAPI before observation by fluorescence microscopy. To analyze intracellular GSH depletion, the intracellular GSH level of the treated cells was analyzed by a GSH kit according to the manufacturer's protocol.

To further investigate the homotypic targeting specificity and intracellular drug release, cells were seeded in a 24-well plate at a density of 3 × 10^4 cells/well and cultured overnight. Then, the cells were incubated with CM@M-MON@Ce6 or M-MON@Ce6 at a final NP concentration of 12.5 μg/mL. After different incubation times, the cells were stained with LysoTracker Green DND-26 and DAPI before observation by fluorescence microscopy. To determine the fluorescence intensity, the cells were washed, trypsinized and resuspended to analyze the fluorescence intensity using FACS. To demonstrate the role of CD47 in the immune escape of

CM@M-MON@Ce6, RAW264.7 cell were pre-treated with anti-CD47, and the cells were incubated with CM@M-MON@Ce6 at a final NP concentration of 12.5 μg/mL. The fluorescence intensity of each group were determined by FACS. All statistical analyses were based on three independent experiments.

For PDT, after 12 h of incubation with the NPs, cells were exposed to light with a wavelength of 660 nm for 5 min (0.15 W/cm^2). For magnetic hyperthermia, after 12 h of incubation with the NPs, cells were exposed to an alternating magnetic field (325 Oe, 262 kHz) for 20 min. For *in vitro* combined therapy, after 12 h of incubation with the NPs, cells were first exposed to an alternating magnetic field (325 Oe, 262 kHz) for 20 min, and the cells were then exposed to light (0.15 W/cm^2) with a wavelength of 660 nm for 5 min. After the treatment, the cell culture medium was then replaced with fresh culture medium, and the cells were incubated for 24 h for subsequent experiments. Cell viability was analyzed by an SRB assay. The apoptotic rate was analyzed by a FITC-Annexin V/dead cell apoptosis kit. To analyze intracellular ROS generation, treated cells were stained with DCFH-DA for 15 min, washed with PBS three times and observed by fluorescence microscopy or analyzed by FACS. The release of HMGB1 in the cell culture supernatant was analyzed by ELISA kit according to the manufacturer's instructions. For analyzing the cell surface expression of calreticulin (CRT), cells were then stained with FITC-labeled anti-CRT and analyzed by FACS. To investigate DC maturation *in vitro*, BMDCs were collected from the bone marrow of BALB/c mice. MCF-7 cells in each group were co-cultured with immature DC cells in a transwell model for 24 h. After staining with

anti-CD11c-FITC, anti-CD80-APC and anti-CD86-PE, the maturation of DC cells was examined by FACS. All statistical analyses were based on three independent experiments.

In vivo MR imaging and biodistribution: All animal experiment protocols were approved by the Ethics Committee for the Use of Experimental Animals of Jilin University. For the establishment of MCF-7 tumor-bearing nude mice, MCF-7 cells (2×10^6 cells) were mixed with an equal volume of Matrigel and orthotopically injected into the mammary fat pads of female nude Balb/c mice (6-8 weeks of age). When the tumor volume reached approximately 250 mm^3, the mice were intravenously injected with CM@M-MON@Ce6 or M-MON@Ce6 at a dose of 12.5 mg/kg. T2-weighted MR images were obtained using a 3.0 T MRI system (Bruker NMR, Germany) at 6 and 24 h after injection using the following parameters: 2800 ms repetition time (TR), 30 ms echo time (TE), 100×100 mm field of view (FOV), 256×256 matrix, and 1 mm slice thickness. For tumor targeting and biodistribution analyses, mice in each group (n=5) were sacrificed at 24 h postinjection, and the livers, spleens, kidneys, hearts, lungs, and tumors were removed, weighed and homogenized, and the fluorescence intensity was determined to analyze the biodistribution. To investigate the pharmacokinetics of the NPs, blood samples were obtained at 5 and 30 min and at 1, 3, 6, 12, 24 and 48 h following injection, and fluorescence analysis of Ce6 was conducted. Pharmacokinetic parameters were calculated and fit to a two-compartment model and a one-way nonlinear model.

In vivo anticancer studies: When the tumor volume reached approximately ~80 mm^3, MCF-7 tumor-bearing nude mice were randomly divided into eight groups (n=5 in each group). The mice were treated with saline, CM@M-MON@Ce6, free Ce6 with light irradiation, CM@M-MON@Ce6 with light irradiation, CM@M-MON@Ce6 with ACMF, CM@M-MON@Ce6+ACMF, CM@M-MON@Ce6+Laser+ACMF, CM@M-MSN@Ce6+Laser+ACMF, M-MON@Ce6+Laser+ACMF via tail vein injection of the NPs (12.5 mg/kg) every 4 days. In this experiment, MCF-7 cell-derived CM-cloaked M-MON@Ce6 were used. For ACMF treatment, mice were placed into a copper coil, and an ACMF (325 Oe, 262 kHz, 20 min) was applied at 11 h postinjection. For PDT treatment, mice were treated with 660-nm light irradiation (0.15 W/cm^2, 10 min) at 12 h postinjection. The body weights and tumor volumes were accurately obtained every two or three days, and the tumor volume was calculated according to the following equation: $length \times width^2 \times 0.52$. On day 28, the mice in all groups were sacrificed, serum samples were separated, and the tumors were imaged and weighed. Biochemical parameters, including aspartate aminotransferase (AST), alanine aminotransferase (ALT), alkaline phosphatase (ALP), albumin (ALB), blood urea nitrogen (BUN), creatinine (CREA), triglyceride (TG), total cholesterol (TC), were analyzed automatically using Coulter LX2D instrumentation (Beckman, Brea, CA). The main organs, including the liver, spleen, kidneys, heart, and lungs, were collected, weighed, fixed and stained with hematoxylin and eosin (H&E).

Combined ICD-triggered immunotherapy: For developing 4T1 tumor-bearing mice, 4T1 cells (1×10^6) were orthotopically injected into the mammary fat pads of female Balb/c mice (6-8 weeks). When the tumor volume reached approximately ~80 mm^3, 4T1 tumor-bearing mice were randomly divided into eight groups (n=5 in each group). Mice were treated with saline, CM@M-MON@Ce6, free Ce6 with light irradiation, CM@M-MON@Ce6 with light irradiation, CM@M-MON@Ce6 with ACMF, CM@M-MON@Ce6+ACMF, CM@M-MON@Ce6+Laser+ACMF, CM@M-MSN@Ce6+Laser+ACMF, and M-MON@Ce6+Laser+ACMF via tail vein injection of the NPs (12.5 mg/kg) every 4 days. In this experiment, 4T1 cell-derived CM-cloaked M-MON@Ce6were used. For ACMF treatment, mice were placed into a copper coil, and an ACMF (325 Oe, 262 kHz, 20 min) was applied at 11 h postinjection. For PDT treatment, mice were treated with 660-nm light irradiation (0.15 W/cm^2, 10 min) at 12 h postinjection. The body weights and tumor volumes were accurately obtained every two or three days, and the tumor volume was calculated according to the following equation: $length \times width^2 \times 0.52$. After treating the mice for 21 days, all the mice were sacrificed and imaged, followed by excising the tumors and lungs. The tumors were weighed, and metastatic nodules on the lungs were counted. H&E staining was conducted to identify micrometastatic lesions on the lungs.

To evaluate the immunological effects of the combined magnetic hyperthermia-PDT treatment, 4T1 tumor-bearing mice were randomly divided into eight groups (n=5 mice in each group). When the tumor volume reached

approximately ~80 mm^3, the mice were treated with saline, CM@M-MON@Ce6, free Ce6 with light irradiation, CM@M-MON@Ce6 with light irradiation, CM@M-MON@Ce6 with ACMF, CM@M-MON@Ce6+ACMF, CM@M-MON@Ce6+Laser+ACMF, CM@M-MSN@Ce6+Laser+ACMF, and M-MON@Ce6+Laser+ACMF. After 5 days of treatment, the mice were sacrificed, and the primary tumors were collected for immunological evaluations. To evaluate the immune cells in the primary tumors, tumors collected from different groups of mice were cut into small pieces and homogenized in PBS (pH 7.4) containing 0.5% FBS to obtain suspensions of single cells, which were then stained with corresponding antibodies after the removal of red blood cells (RBC) using RBC lysis buffer (Solarbio). To identify CTLs (CD3+CD4-CD8+) and regulatory T cells (Treg cells) (CD3+CD4+Foxp3+), the following antibodies (from eBioscience, Biolegend) were used: CD45 monoclonal antibody (30-F11, PE-Cyanine7), CD3e monoclonal antibody (145-2C11, APC), CD4 monoclonal antibody (GK1.5, Alexa Fluor 488), CD8a monoclonal antibody (53-6.7, PE), True-Nuclear™ Mouse Treg Flow™ Kit (FOXP3 Alexa Fluor® 488/CD4 APC/CD25 PE), and TruStain FcX™ (anti-mouse CD16/32). The antibodies were used according to the manufacturer's protocols. All data were collected by flow cytometer and analyzed with FlowJo software. Additionally, serum samples were collected from the mice to analyze the levels of HMGB1, TNF-α, IFN-ɣ, and IL-6 through ELISA.

For checkpoint blockade immunotherapy combined with magnetic hyperthermia-PDT, 4T1 tumor-bearing mice were randomly divided into six groups

(n=5 mice in each group). When the tumor volume reached approximately ~80 mm^3, the mice were treated with saline, α-CTLA-4, CM@M-MON@Ce6+Laser, CM@M-MON@Ce6+ACMF, CM@M-MON@Ce6+Laser+ACMF, CM@M-MON@Ce6+Laser+α-CTLA-4, CM@M-MON@Ce6+ACMF+α-CTLA-4, and CM@M-MON@Ce6+Laser+ACMF+α-CTLA-4 via tail vein injection of the NPs (12.5 mg/kg) every 4 days. For the checkpoint blockade immunotherapy group, anti-CTLA-4 was administered every 4 days by intraperitoneal injection at a dose of 20 μg per mouse after 12 hours of combined magnetic hyperthermia-PDT treatment following the above-mentioned protocol. The body weights and tumor volumes were accurately obtained every two or three days, and the tumor volume was calculated according to the following equation: $\text{length}\times\text{width}^2\times0.52$. At day 9 posttreatment, serum samples were collected from the mice to analyze the levels of TNF-α, IFN-ɣ, and IL-6. Furthermore, tumor-infiltrating lymphocytes in the metastatic tumors were evaluated. After 21 days of treatment, all the mice were sacrificed, and the main organs, including the liver, spleen, kidneys, heart, and lungs, were collected, fixed and stained with H&E. The lung tissues were imaged, and metastatic nodules on the lungs were counted. Biochemical parameters, including AST, ALT, ALP, BUN, CREA, ALB, TC, and TGs, were analyzed automatically using Coulter LX2D instrumentation (Beckman, Brea, CA).

Statistical analysis: All the results are presented as the mean ± standard deviation (S.D.). Differences between groups were analyzed using Student's t-test. Differences among more than two groups were analyzed using one-way analysis of variance, and

the Bonferroni post hoc test was used to analyze differences between any two groups.

$P < 0.05$ was considered to indicate a statistically significant difference.

Reference:

[1] H. Xia, L. Zhang, Q.-D. Chen, L. Guo, H.-H. Fang, X.-B. Li, J.-F. Song, X.-R. Huang, H.-B. Sun, *J. Phys. Chem. A.* **2009,** *113*, 18542.
[2] Z. Wang, Z. Chang, M. Lu, D. Shao, J. Yue, D. Yang, X. Zheng, M. Li, K. He, M. Zhang, *Biomaterials* **2018**, *154*, 147.
[3] Z. Chang, Z. Wang, D. Shao, J. Yue, M. Lu, L. Li, M. Ge, D. Yang, M. Li, H. Yan, *Sens. Actuators. B. Chem.* **2018,** *260*, 1004.
[4] D. Shao, M. Li, Z. Wang, X. Zheng, Y. H. Lao, Z. Chang, F. Zhang, M. Lu, J. Yue, H. Hu, *Adv. Mater.* **2018,** *30*, 1801198.

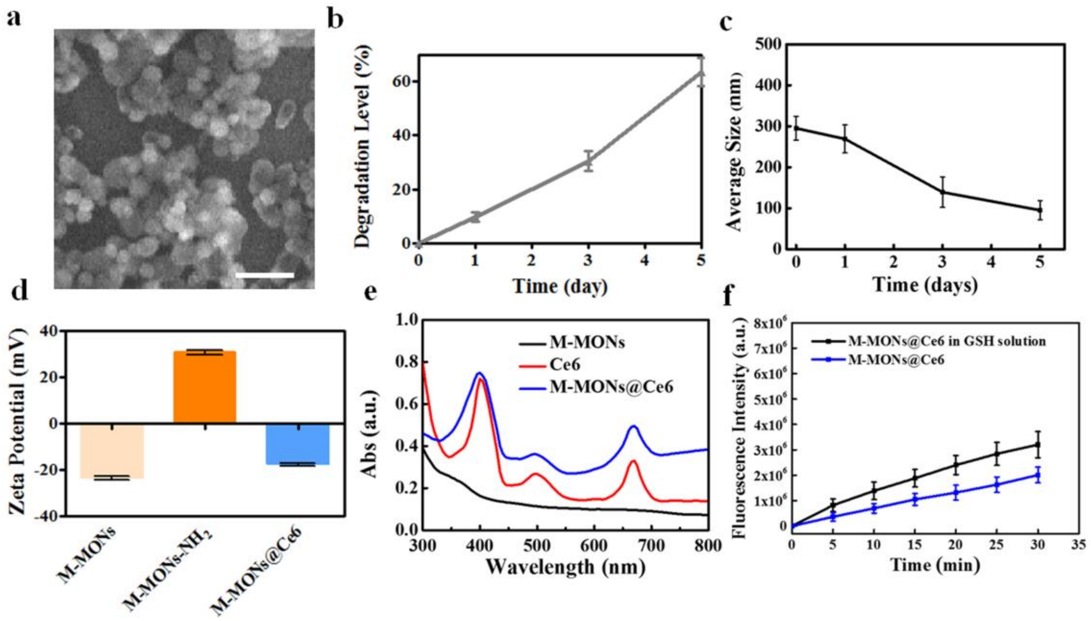

Figure S1. Characterization of M-MON@Ce6. (a) Scanning electron microscopy (SEM) images of M-MONs, (b) Degradation level and (c) Average size of M-MONs after incubation with 5 mM GSH. (d) zeta potentials, (e) UV-vis extinction spectra of M-MONs and M-MON@Ce6. (f) Time-dependent SOSG fluorescence in M-MON@Ce6 solutions and GSH-treated M-MON@Ce6 for 24 h. These data represent three separate experiments. Mean values ± SD.

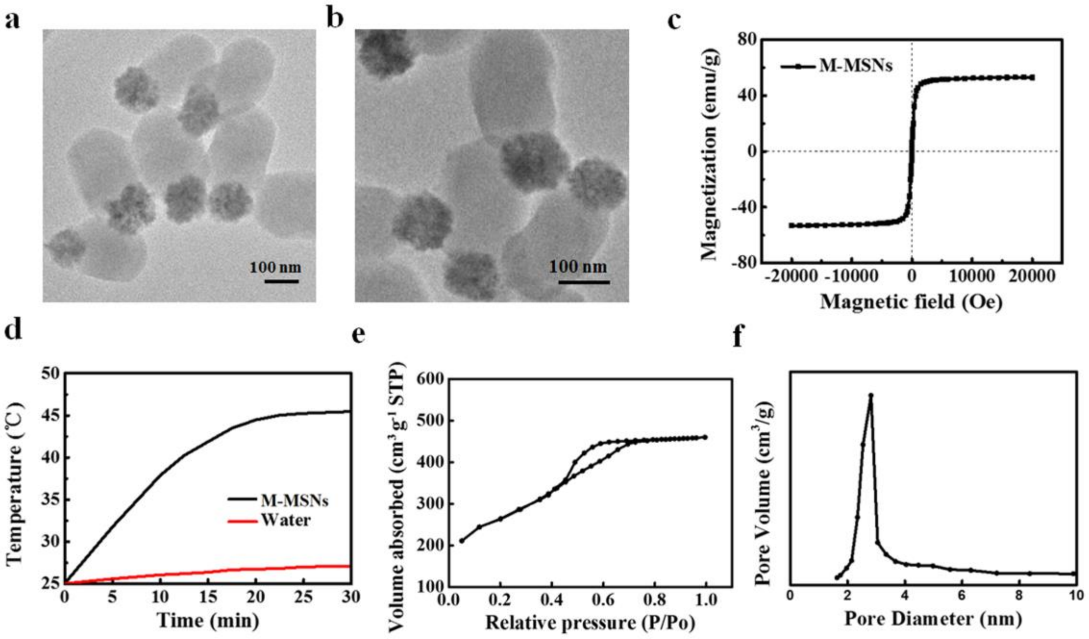

Figure S2. Characterization of M-MSN@Ce6. TEM images of M-MSNs at (a) 0 and (b) 7 days of incubation in 5 mM GSH solution. (c) The magnetization curve, (d) temperature-time curves, (e) N_2 sorption isotherms, and (f) pore size distribution of M-MSNs.

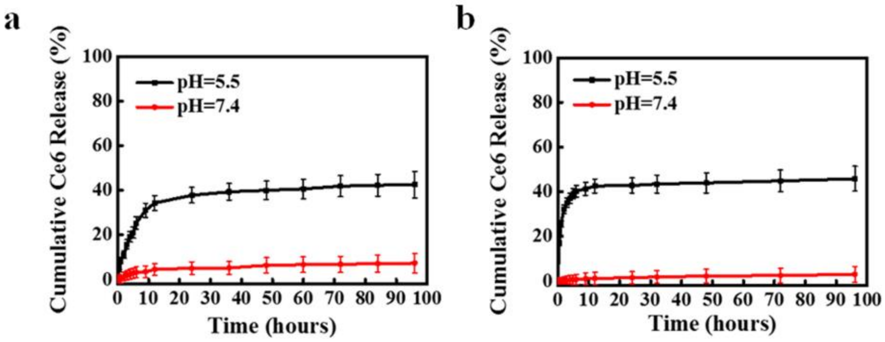

Figure S3. Drug release profiles of Ce6@M-MSNs in (a) 5 mM GSH (pH=7.4 and 5.5) and (b) 0 mM GSH (pH=7.4 and 5.5). The data are presented as the mean ± S.D. (n = 3).

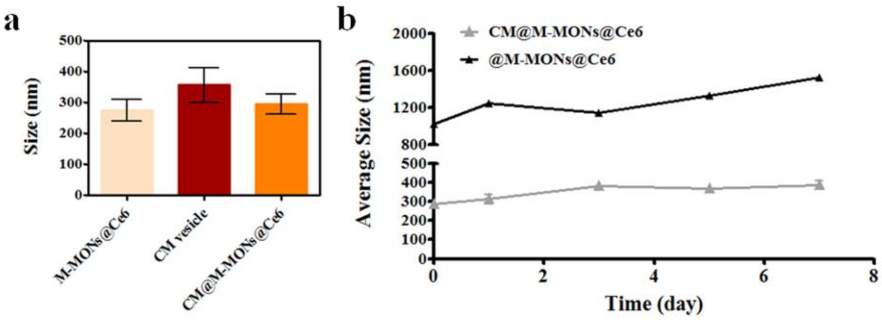

Figure S4. (a) The size distribution of Ce6@M-MONs and CM@Ce6@M-MONs in water. (b) Time-dependent colloidal stability of Ce6@M-MONs and CM@Ce6@M-MONs in PBS.

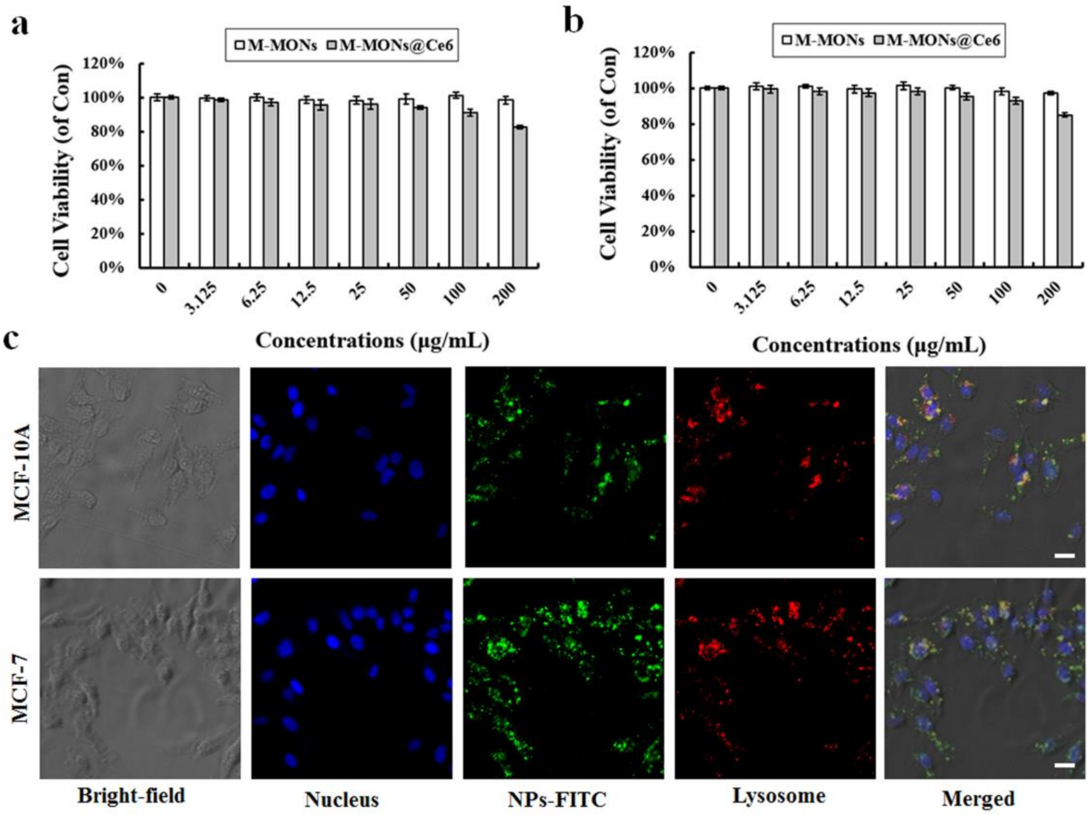

Figure S5. The cytotoxicity of M-MONs@Ce6 at various concentrations in (a) MCF-7 and (b) MCF-10A cells after 24 h of incubation. The data are from three separate experiments and are presented as the mean ± S.D. (c) Fluorescence images of MCF-7 and MCF-10A cells incubated with FITC-labeled M-MONs for 3 h. The green fluorescence indicates the location of the FITC-NPs. The lysosomes/endosomes were stained with Lysotracker Red (red), and the cell nuclei were stained with DAPI (blue). The scale bars indicate 10 μm.

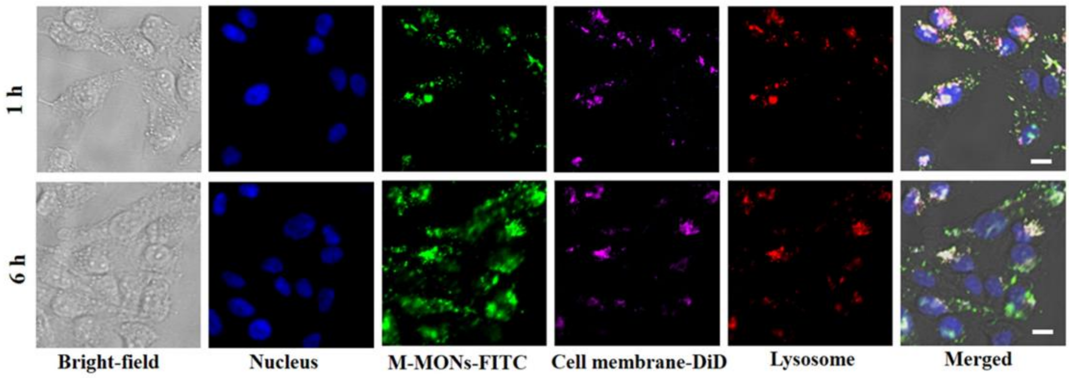

Figure S6. Intracellular colocalization of DiD-labeled cancer cell ghost membranes and FITC-labeled M-MONs in MCF-7 cells after 1 and 6 h. The scale bars indicate 5 μm.

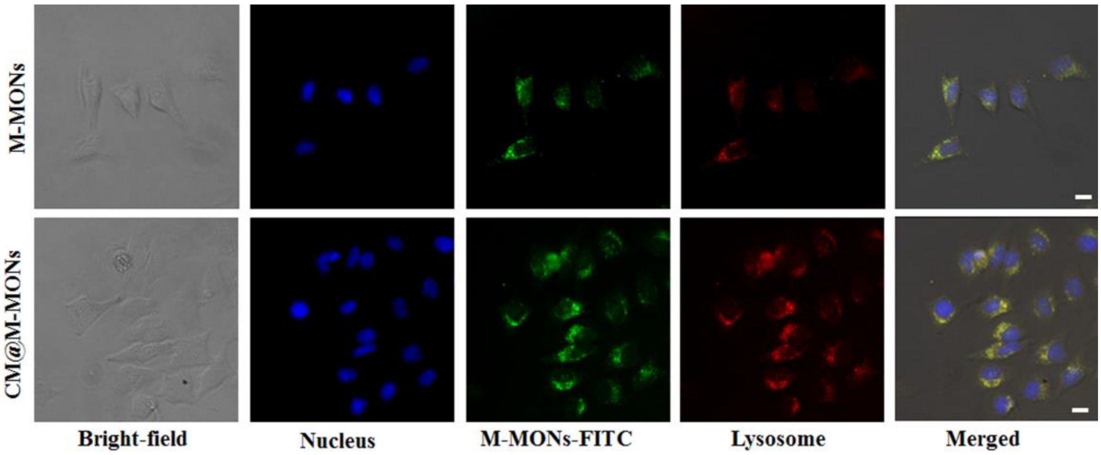

Figure S7. Fluorescence images of MCF-7 cells incubated with DiD-labeled cancer cell membrane-cloaked FITC-labeled M-MONs for 2 h. The green fluorescence indicates the location of the FITC-NPs. The cancer cell membrane shells were stained with DiD (red), and the cell nuclei were stained with DAPI (blue). The scale bars indicate 10 μm.

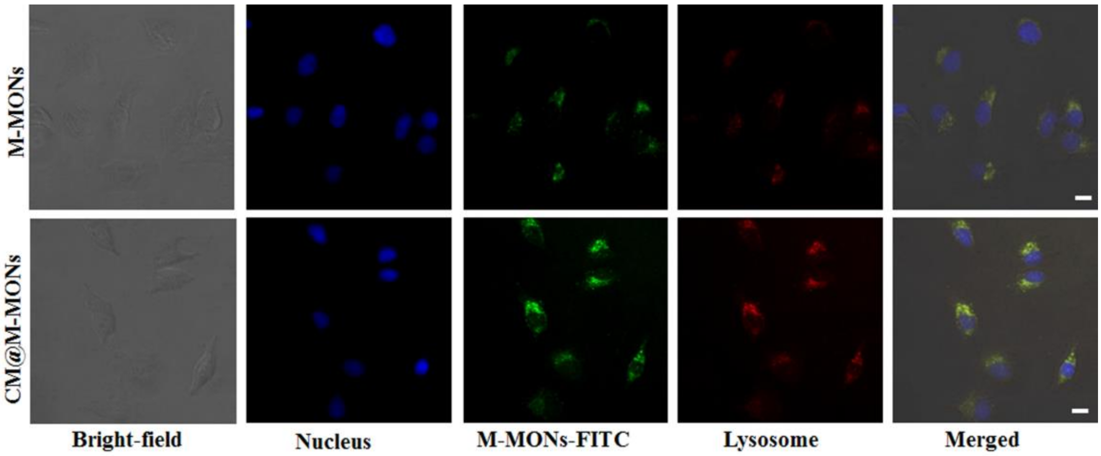

Figure S8. Fluorescence images of MCF-10A cells incubated with DiD-labeled cancer cell membrane-cloaked FITC-labeled M-MONs for 2 h. The green fluorescence indicates the location of the FITC-NPs. The cancer cell membrane shells were stained with DiD (red), and the cell nuclei were stained with DAPI (blue). The scale bars indicate 10 μm.

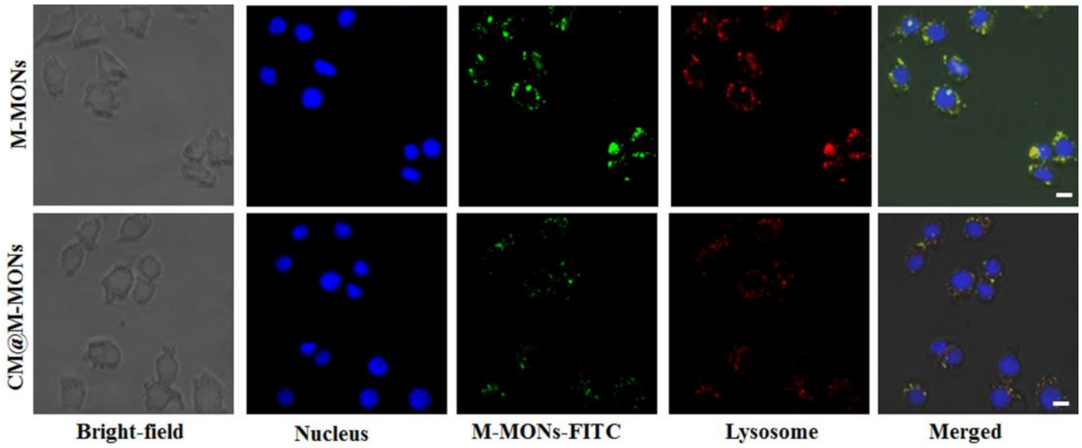

Figure S9. Fluorescence images of RAW 264.7 cells incubated with DiD-labeled cancer cell membrane-cloaked FITC-labeled M-MONs for 2 h. The green fluorescence indicates the location of the FITC-NPs. The cancer cell membrane shells were stained with DiD (red), and the cell nuclei were stained with DAPI (blue). The scale bars indicate 10 μm.

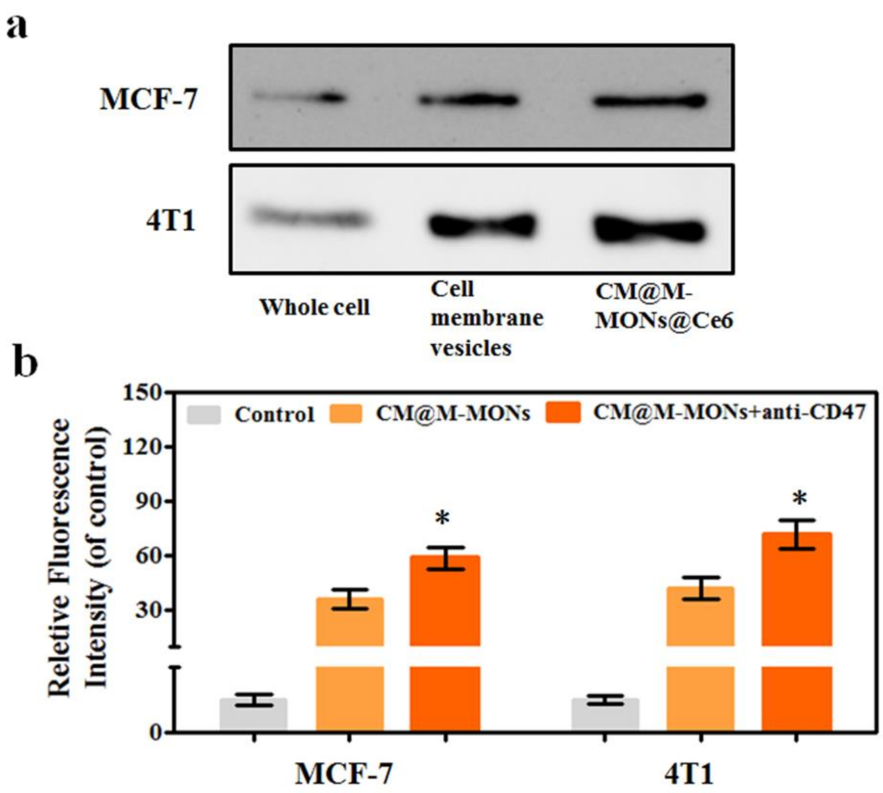

Figure S10. (a) CD47 characterization by Western blotting analysis of CM@M-MON@Ce6. (b) The relative fluorescence intensity of RAW264.7 cells after incubation with CM@FITC-M-MONs with or without anti-CD47 for 6 h. The data are presented as the mean ± S.D. (n = 3). $^{*}p < 0.05$ compared with the M-MON group.

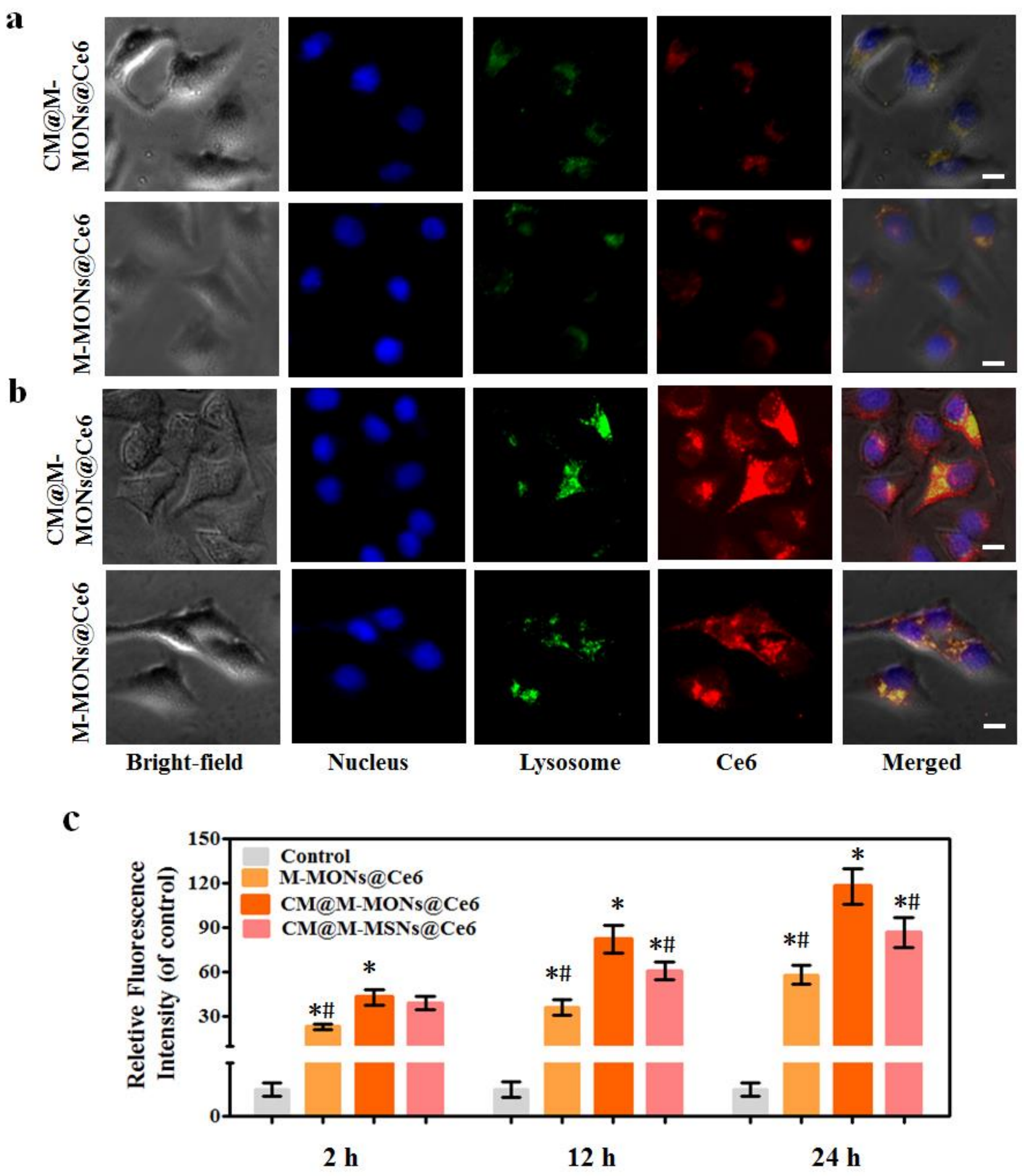

Figure S11. Fluorescence images of MCF-7 cells incubated with Ce6@M-MONs and CM@Ce6@M-MONs for (a) 2 and (b) 24 h. The green fluorescence indicates the location of the lysosome, and the cell nuclei were stained with DAPI (blue). The scale bars indicate 10 μm. (c) The relative fluorescence intensity of MCF-7 cells after incubation with CM@M-MON@Ce6 for 2, 12 and 24 h. The data are presented as the mean ± S.D. (n = 3). $^{*}p < 0.05$ compared with the Ce6 group and $^{\#}p < 0.05$ compared with the CM@M-MON@Ce6 group.

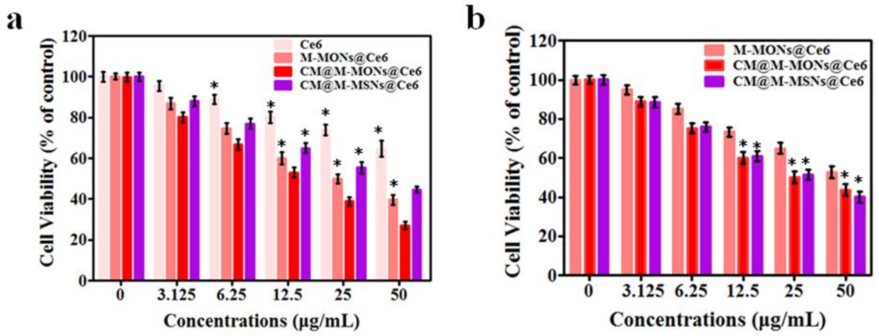

Figure S12. (a) Cell viability of MCF-7 cells incubated with various concentrations of CM@M-MON@Ce6 for 12 h, exposed to laser irradiation (660 nm, 2 W/cm^2) for 5 min and incubated for an additional 24 h. The data are presented as the mean ± S.D. (n = 3). $^*p < 0.05$ compared with the CM@M-MON@Ce6 group. (b) Cell viability of MCF-7 cells treated with various concentrations of CM@M-MON@Ce6 for 12 h, exposed to an ACMF for 20 min and incubated for an additional 24 h. The data are presented as the mean ± S.D. (n = 3). $^*p < 0.05$ compared with the M-MON@Ce6 group.

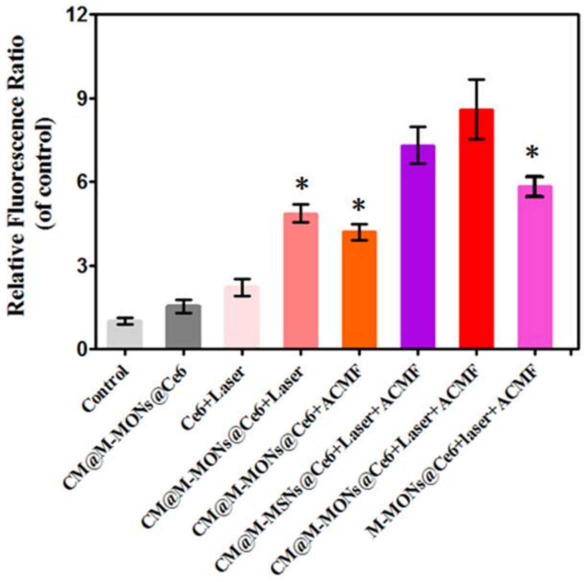

Figure S13. MCF-7 cells were incubated with CM@M-MON@Ce6 (12.5 μg/mL) for 2 h, followed by a 20 min exposure to an ACMF or/and a 5 min of exposure to laser irradiation with a 20-min exposure to an ACMF. The intracellular ROS fluorescence intensity was determined by FACS after 6 h of exposure. The data are presented as the mean ± S.D. (n = 3). $^{*}p < 0.05$ compared with the CM@M-MON@Ce6+laser+ACMF group.

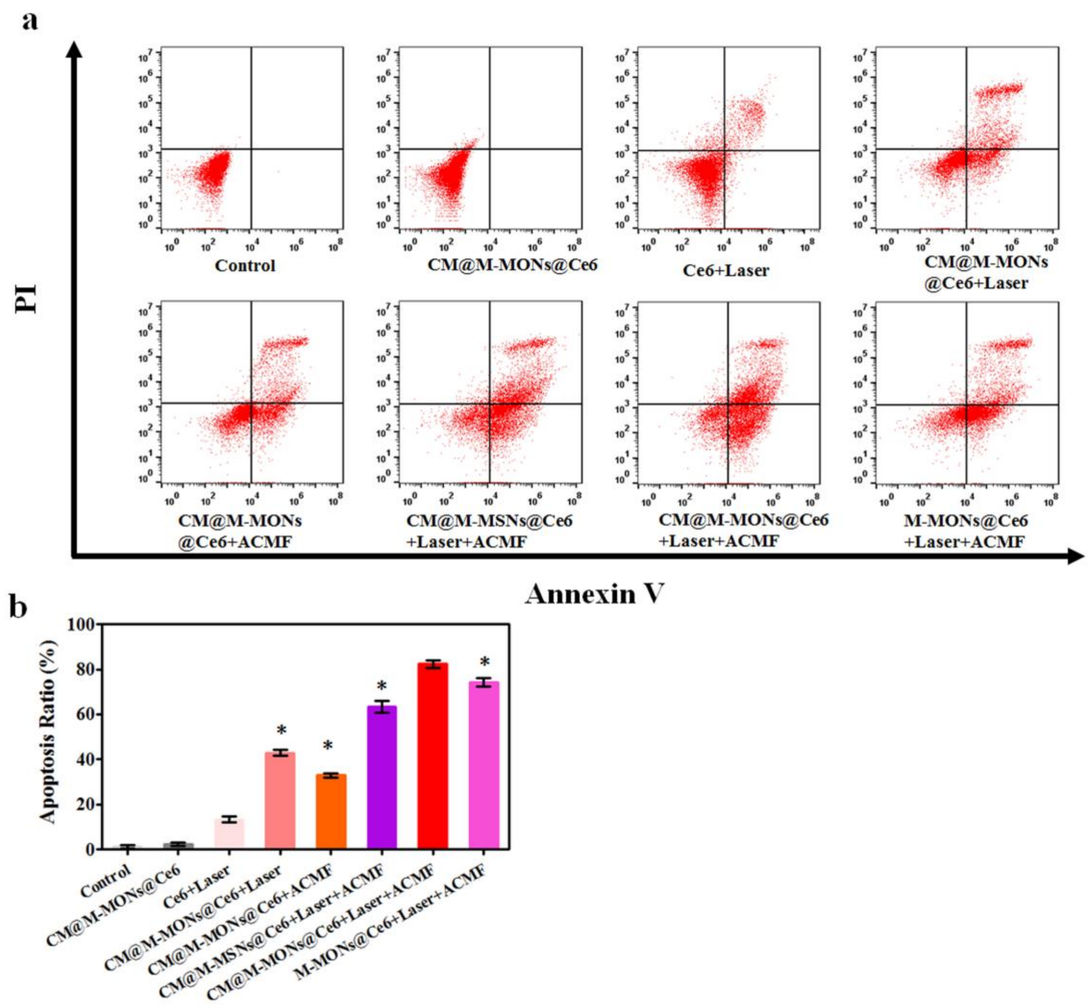

Figure S14. (a) MCF-7 cells were incubated with CM@M-MON@Ce6 (12.5 μg/mL) for 2 h, followed by a 20 min exposure to an ACMF or/and a 5 min of exposure to laser irradiation with a 20 min exposure to an ACMF. Apoptosis was analyzed by FACS after 6 h of exposure. (b) Corresponding apoptosis ratio after 24 h of exposure. The data are presented as the mean ± S.D. (n = 3). $^{*}p < 0.05$ compared with the CM@M-MON@Ce6+Laser+ACMF group.

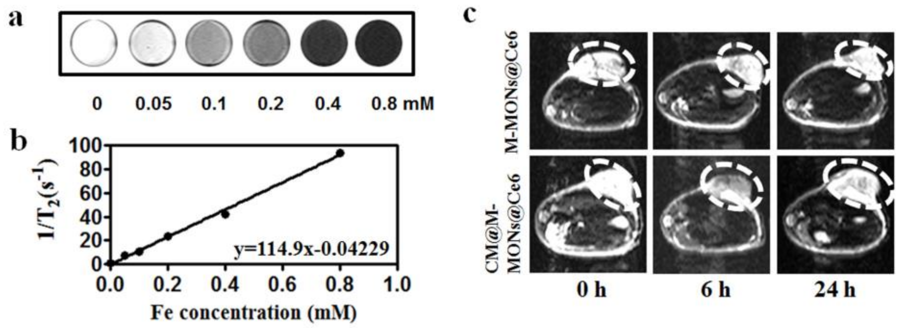

Figure S15. (a) MR images of CM@M-MON@Ce6 with different concentrations of Fe. (b) Plots of the inverse transverse relaxation time ($1/T_2$) versus the Fe concentration of the CM@M-MON@Ce6. (c) *In vivo* T_2-weighted images of MCF-7 tumor-bearing mice at 6 and 24 h after the intravenous injection of CM@M-MON@Ce6 (12.5 mg/kg).

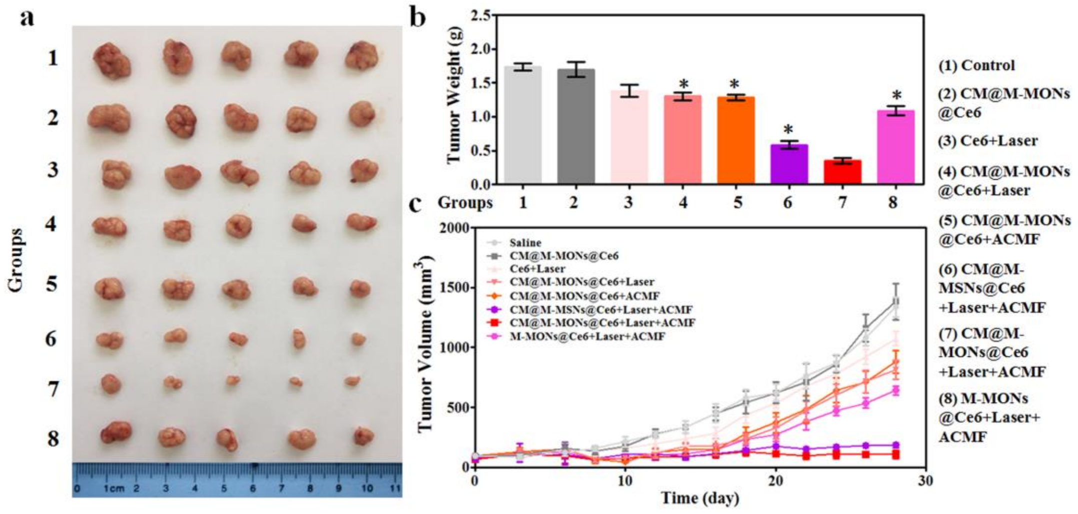

Figure S16. (a) Representative tumor images, (b) tumor weights, and (c) tumor volumes of the MCF-7 tumor-bearing nude mice from each group over 28 days. The data are presented as the mean ± S.D. (n = 5). *$p < 0.05$ compared with the CM@M-MON@Ce6+laser+ACMF group.

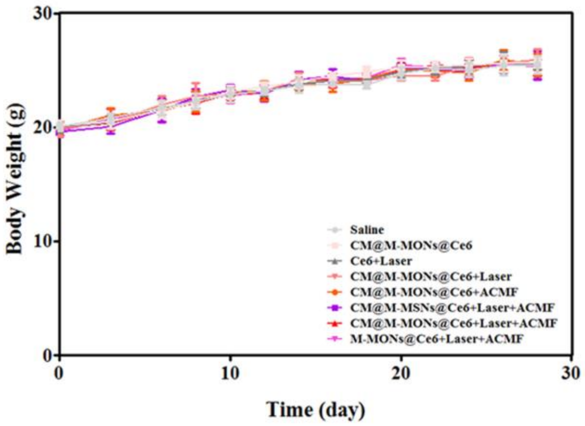

Figure S17. The body weights of the MCF-7 tumor-bearing mice from each group over 28 days. The data are presented as the mean ± S.D. (n = 5).

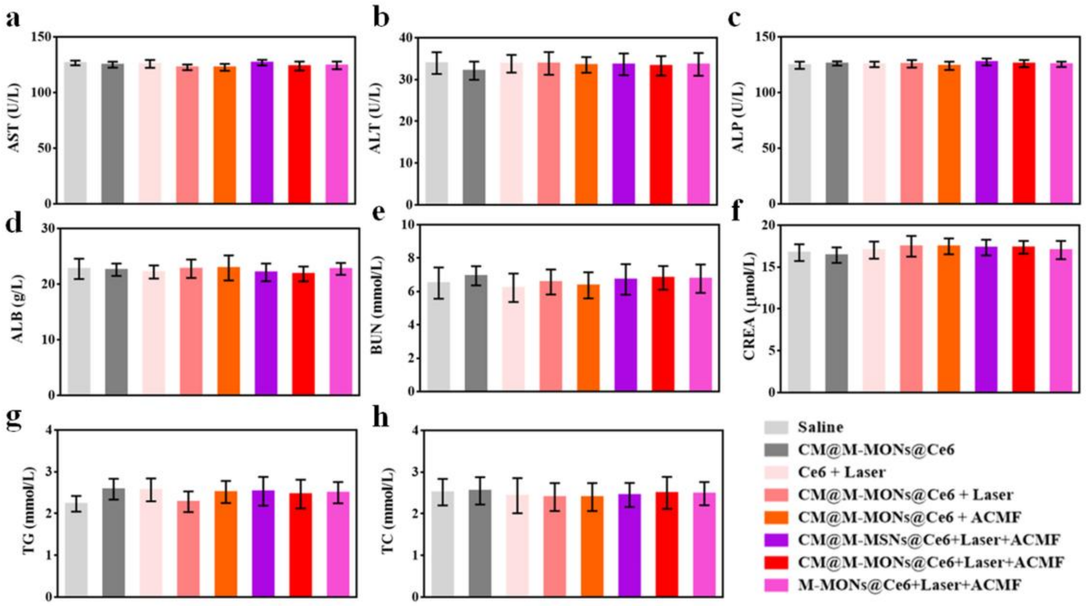

Figure S18. Biochemical parameters, including (a) aspartate aminotransferase (AST), (b) alanine aminotransferase (ALT), (c) alkaline phosphatase (ALP), (d) albumin (ALB), (e) blood urea nitrogen (BUN), (f) creatinine (CREA), (g) triglyceride (TG), (h) total cholesterol (TC), of the MCF-7 tumor-bearing mice after 28 days of treatment. The data are presented as the mean ± S.D. (n=5).

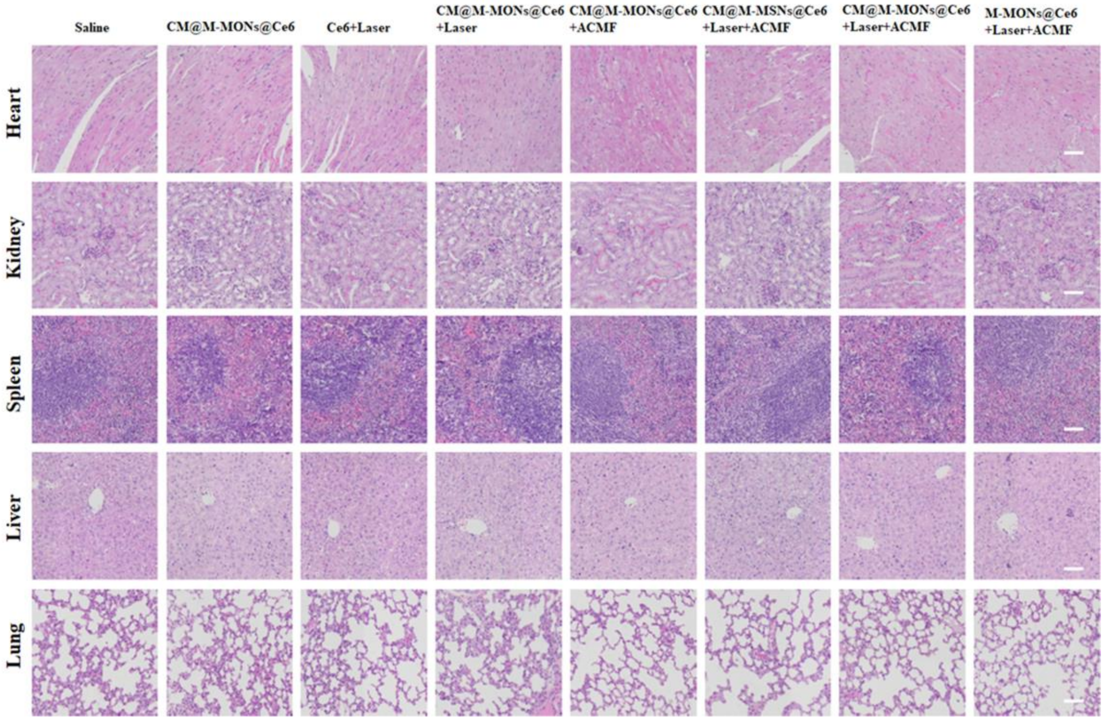

Figure S19. Histological evaluation of the major organs, including the liver, spleen, kidneys, heart, and lungs, of the MCF-7 tumor-bearing nude mice after 28 days of treatment. The scale bars indicate 100 μm.

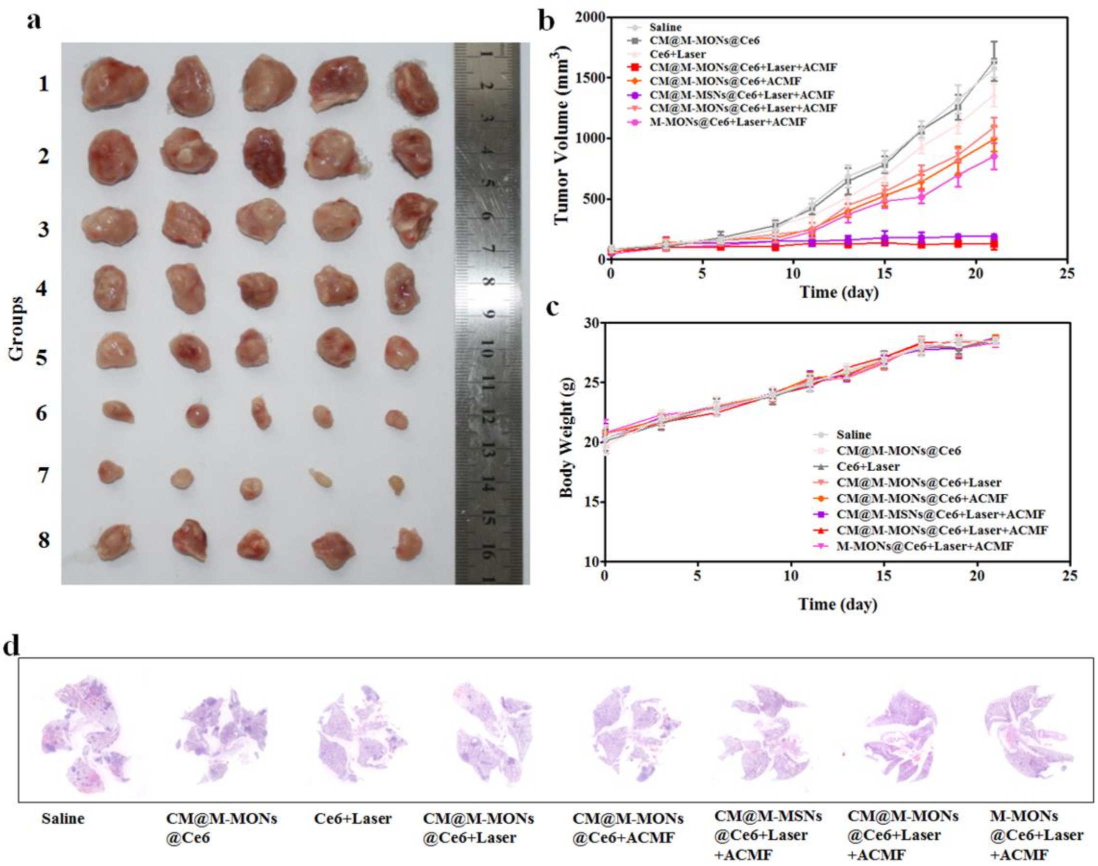

Figure S20. (a) Representative tumor images, (b) tumor volumes, (c) body weights, and (d) representative images of H&E-stained sections of the lungs of the 4T1 tumor-bearing mice from each group over 21 days. The data are presented as the mean ± S.D. (n = 5).

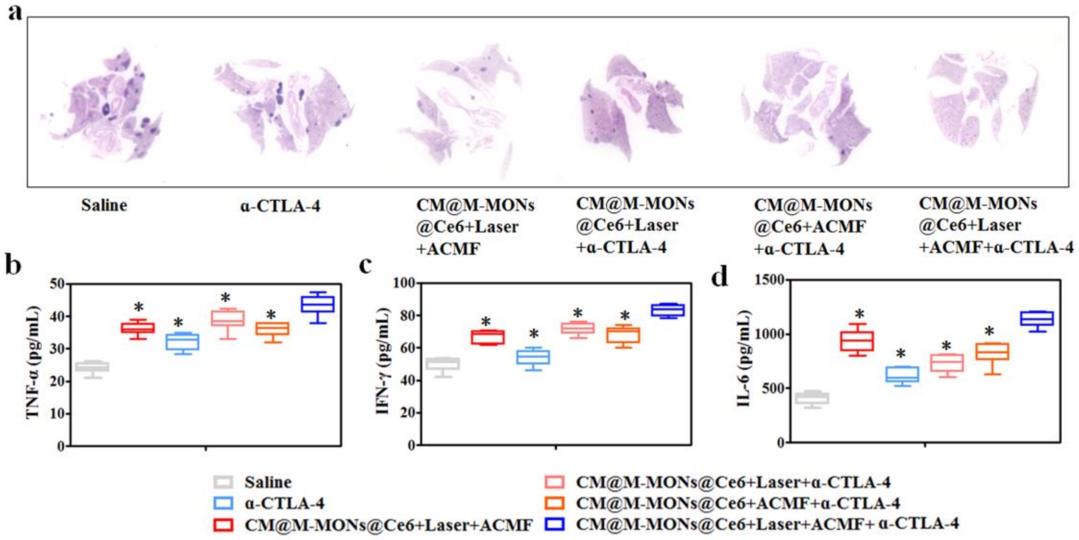

Figure S21. (a) Representative images of H&E-stained sections of the 4T1 tumor-bearing mice from each group over 21 days. (b) TNF-α, (c) IFN-ɣ, and (d) IL-6 in the serum from the lungs of the 4T1 tumor-bearing mice from each group over 9 days. The data are presented as the mean ± S.D. (n = 5). *p < 0.05 compared with the CM@M-MON@Ce6+laser+ACMF+α-CTLA-4 group.

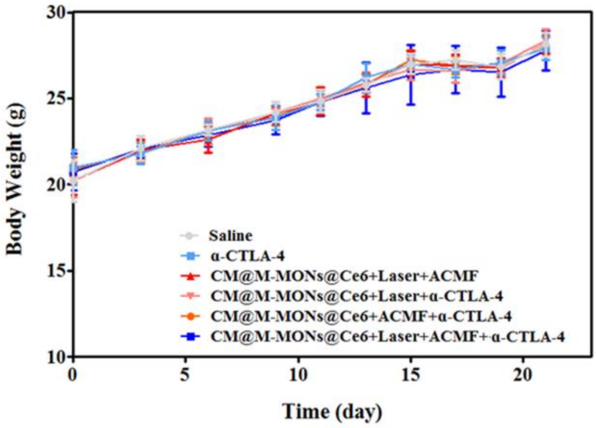

Figure S22. The body weights of the 4T1 tumor-bearing mice from each group over 21 days. The data are presented as the mean ± S.D. (n = 5).

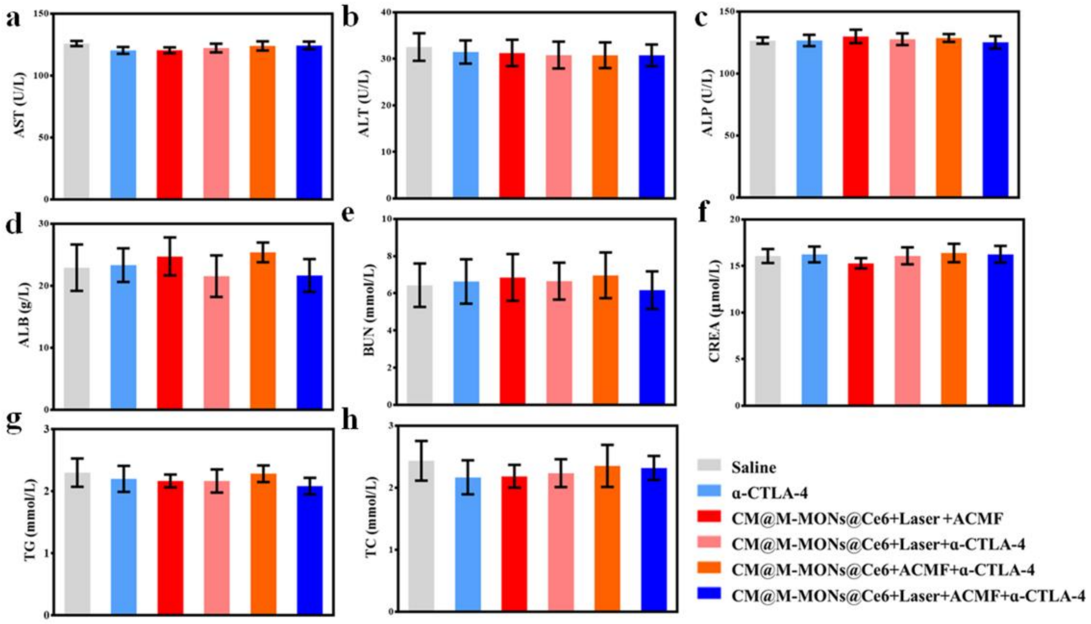

Figure S23. Biochemical parameters, including (a) aspartate aminotransferase (AST), (b) alanine aminotransferase (ALT), (c) alkaline phosphatase (ALP), (d) albumin (ALB), (e) blood urea nitrogen (BUN), (f) creatinine (CREA), (g) triglyceride (TG), (h) total cholesterol (TC), of the 4T1 tumor-bearing mice after 21 days of treatment. The data are presented as the mean ± S.D. (n=5).

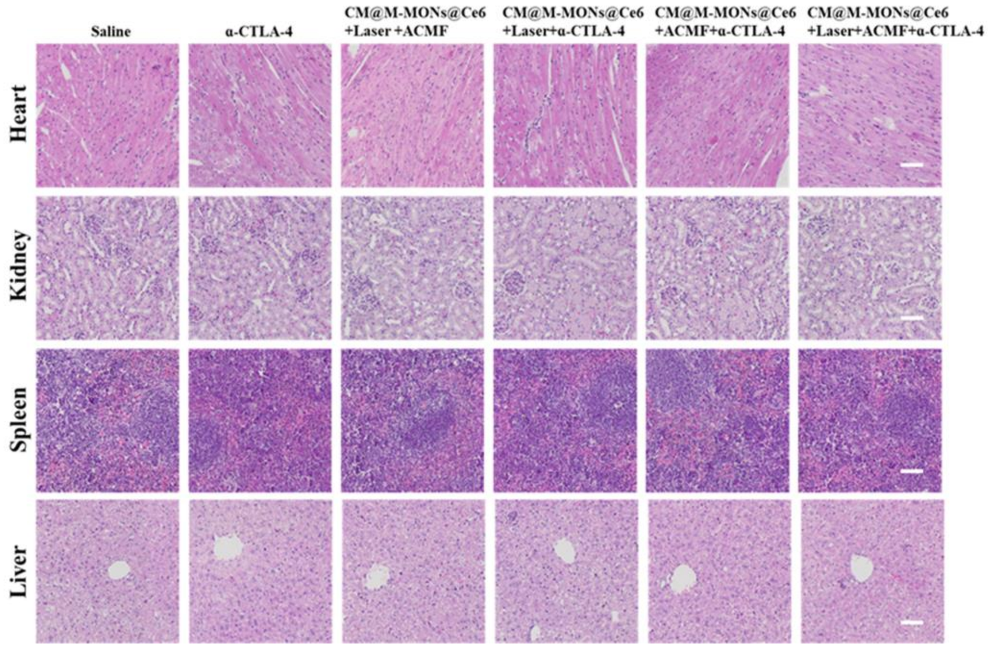

Figure S24. Histological evaluation of the major organs, including the liver, spleen, kidneys, heart, and lungs, of the 4T1 tumor-bearing nude mice after 21 days of treatment. The scale bars indicate 100 μm.
